# Computational Methods for Estimating Molecular System from Membrane Potential Recordings in Nerve Growth Cone

**DOI:** 10.1038/s41598-018-22506-3

**Published:** 2018-03-14

**Authors:** Tatsuya Yamada, Makoto Nishiyama, Shigeyuki Oba, Henri Claver Jimbo, Kazushi Ikeda, Shin Ishii, Kyonsoo Hong, Yuichi Sakumura

**Affiliations:** 1 Graduate School of Information Science, Nara Institute of Science and Technology, Nara, Japan; 2 Department of Biochemistry, New York University School of Medicine, New York, USA; 3 Graduate School of Informatics, Kyoto University, Kyoto, Japan; 4 Graduate School of Biological Sciences, Nara Institute of Science and Technology, Nara, Japan; 5 KASAH Technology, Inc, New York, USA; 6 School of Information Science and Technology, Aichi Prefectural University, Aichi, Japan

## Abstract

Biological cells express intracellular biomolecular information to the extracellular environment as various physical responses. We show a novel computational approach to estimate intracellular biomolecular pathways from growth cone electrophysiological responses. Previously, it was shown that cGMP signaling regulates membrane potential (MP) shifts that control the growth cone turning direction during neuronal development. We present here an integrated deterministic mathematical model and Bayesian reversed-engineering framework that enables estimation of the molecular signaling pathway from electrical recordings and considers both the system uncertainty and cell-to-cell variability. Our computational method selects the most plausible molecular pathway from multiple candidates while satisfying model simplicity and considering all possible parameter ranges. The model quantitatively reproduces MP shifts depending on cGMP levels and MP variability potential in different experimental conditions. Lastly, our model predicts that chloride channel inhibition by cGMP-dependent protein kinase (PKG) is essential in the core system for regulation of the MP shifts.

## Introduction

Estimation or determination of unknown functions or targets by indirect measurements, e.g. the estimation of the DNA double helix from its X-ray diffraction pattern^1^, the functional connectivity of neurons from the activation pattern^2^, the identification of cell type from the gene expression pattern^3^, and cancer diagnosis from breath gas components^4^ have been demonstrated. We present a computational derivation for the estimation of bimolecular interactions from an observed time series of electrophysiological activities recorded from nerve growth cones.

Axon guidance is essential for establishing a neuronal network during nervous system development^5,6^. The biomolecular signaling pathways that instruct the direction of a navigating growth cone have been intensively investigated^7–10^. Many studies^11–13^, including our’s^8,14^, have shown that the second messenger, cGMP, is a downstream effecter of the guidance cue, Sema3A. A growth cone normally exhibits a repulsive response to a Sema3A gradient^11,15^. However, this repulsion converts to attraction if the intracellular cGMP level is elevated^8,16^. Our studies revealed that the growth cone turning direction depends on the state of the growth cone membrane potential (MP); a hyperpolarized or depolarized state induces, respectively, either repulsion or attraction in response to many diffusible guidance molecules^16^. Furthermore, it has been shown that a low level of cGMP causes growth cone hyperpolarization, whereas a high level of cGMP causes depolarization^16^, demonstrating that a cGMP signal regulates the MP shifts, which determine the growth cone turning direction. The signaling cascade that converts the guidance cue-induced biomolecular system to electrical signals, however, remains largely unknown.

To computationally estimate the biomolecular network responsible for axon guidance from growth cone MP recordings, three major hurdles must be overcome: 1. the limited availability of the recording data due to the amount of labor required; 2. the large cell-to-cell variability^17–19^, which affects the observed MP; 3. multiple unknown factors that potentially cooperate to regulate the molecular network. The current computational study considers the limited availability of data by utilizing each MP time series (MPTS) that contain over 10,000 data points within one recording sample, thereby providing sufficient data points to perform quantitative computational analysis and to fit a deterministic model to a cell-dependent characteristic. To extract a reliable estimation of a biomolecular network from the small sample size of MP data sets, we applied Bayesian a reverse-engineering framework^20,21^ that has been an especially effective method for studying small number data sets and has been successfully applied in many neuroscience studies^2,3,22–24^. Briefly, by the Bayesian reverse-engineering framework of the system comprised of different physical quantities, we computed the posterior distributions of the parameters that are derived from a fitness of the deterministic biochemical reaction model developed using the experimental MP data sets and prior constraints. Second, the computational study considers the cell-to-cell variability by expressing it as probability distributions of the model parameters^18,20,21,25^. Lastly, the study considers the involvement of multiple unknown factors in the signaling pathway by developing a signaling cascade-based model that simplifies the multiple bio-molecular cascades, and introduced MPTSs data sets into the model.

Many studies have utilized the Bayesian framework to deduce potential unknown biochemical interactions from direct biochemical reaction measurements^18,19,26–28^. We present a novel approach that estimates the molecular signaling pathway from electrophysiological recordings. The current study addresses the system uncertainty and cell-to-cell variability by applying parameter categorization to separate the model parameters into two categories: cell-common core system and cell-dependent peripheral properties. To select the most plausible molecular pathway from multiple model candidates, we applied the Bayesian evidence^29,30^ that satisfies model simplicity while considering all possible parameter ranges. Our combined Bayesian reverse-engineered framework with a mathematical model approach successfully reproduces the quantitative dependency of steady-state MP shifts based on cGMP levels, and reveals that chloride channel inhibition by PKG is essential for MP shifts. Thus, we provide a novel computational methodology to estimate the essential molecular signaling components in transducing the electrical responses elicited during growth cone turning.

## Results

### A mathematical model of cGMP signaling inducing growth cone membrane potential shifts

**Figure 1:**
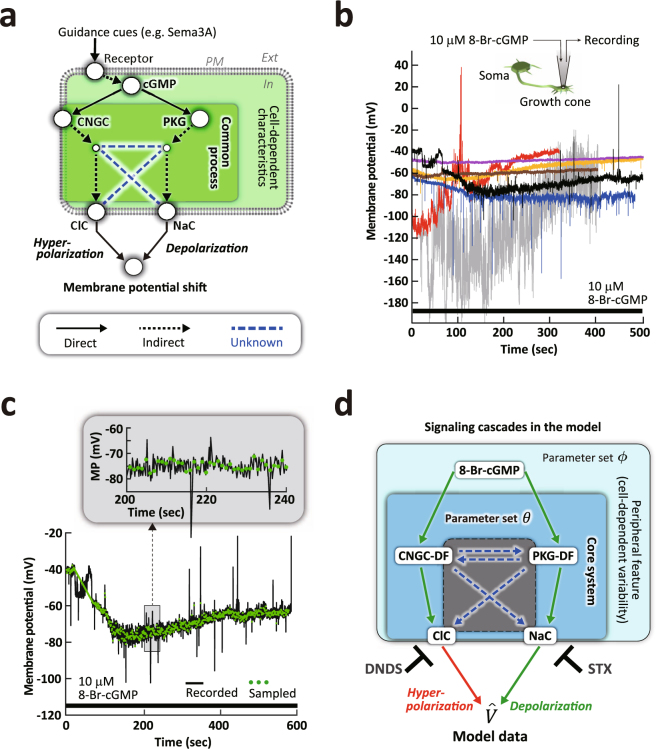
Conversion of a biomolecular signal to an electrophysiological signal in a nerve growth cone. (**a**) Signaling pathways from an extracellular guidance cue, e.g. Sema3A, to membrane potential (MP) shifts in a growth cone. Sema3A, upon binding to its receptor on the plasma membrane (PM), increases the intracellular cGMP level. The effector proteins, Cyclic nucleotide-gated ion channels (CNGC) and cGMP-dependent protein kinases (PKG) that directly bind to cGMP (solid arrows) trigger, respectively, the activation of chloride channels (ClC) and sodium channels (NaC), which induce either hyperpolarization or depolarization. The potential cross interactions between the downstream effectors, which are unknown, are indicated by dashed blue lines. (**b**) Growth cone recording stimulated by a stimulant, 10 μM 8-Br-cGMP, a membrane permeable cGMP analogue (Top). Samples of recorded growth cone MP time series (MPTS; control: n = 7). (**c**) The MPTS contains over 10,000 data points (black). Outlier noises, such as spikes, are removed by sampling the data points at a 1 sec interval (green dots; see upper inset, enlargement of dashed square region; Supplementary Fig. 1). (**d**) The schematic diagram illustrates the mathematical model that functions as an alternative system and is designed in mesoscopic scale that expresses effective molecular signal flows. The control model contains the signaling pathway from the cGMP stimulation to MP shifts, via CNGC- and PKG-downstream factors (DFs), corresponding to the pathways in (**a**). The unknown signal flows between CNGC-DF, PKG-DF, ClC, and NaC in the core system are indicated within the dashed grey box. The parameters of the unknown flows and known factors, i.e., CNGC-DF to ClC and PKG-DF to NaC, are involved in the blue box and characterized by the core system parameter set, $\theta$. The 8-Br-cGMP stimulation and MP, which are outside of the core system, are characterized by cell-dependent peripheral parameter set, $\varphi$. We modeled the diversified data as a core system with peripheral elements representing cell-to-cell variability. Two pharmacological experimental conditions were also modeled, which represent complete inhibition of ClC, blocked by DNDS (n = 5) and NaC, blocked by STX (n = 4) (see Supplementary Fig. 2). A total of 16 datasets (control: n = 7; DNDS: n = 5; STX: n = 4) were analyzed.

The induction of membrane potential (MP) shifts, i.e., hyperpolarization to depolarization and vice versa by cGMP signaling, involves multifaceted molecular, biochemical, and biophysical processes in the growth cone (Fig. 1a). Diffusible guidance molecules, such as Sema3A, increase the intracellular cGMP level, and depending on the level of increase, either cGMP-mediated hyperpolarization (at low cGMP level) or PKG-mediated depolarization (at high cGMP level) occurs, which subsequently causes, respectively, growth cone repulsion or attraction^16^. However, how hyperpolarizing and depolarizing channel activities are regulated in response to different levels of a cGMP signal that regulates the bidirectional MP shifts is largely unknown.

We used the abundant data points from MP recordings from *Xenopus* spinal neuron growth cones (Fig. 1b; see Methods). The time series of each MP (MPTS) recorded during 10 minutes in response to stimulation by 8-Br-cGMP (a membrane permeable cGMP analogue injected into the growth cone by a recording pipette) is comprised of more than 10,000 data points (see Fig. 1b). We eliminated the noise, such as spikes, by sampling the recorded MPTS at 1 sec intervals (green dots in Fig. 1c, Supplementary Fig. 1). The sampled MPTS provides a sufficient number of data points (several hundred) to allow us to develop a quantitative and deterministic mathematical model to dissect the cGMP signaling responsible for inducing growth cone MP shifts. Our model incorporates the mesoscopic molecular signal flows within a core system of the model with parameter set, $\theta$, that regulate chloride and sodium channels (ClC and NaC), respectively, hyperpolarizing and depolarizing channels that regulate MP shifts (Fig. 1d). To extract a partial subsystem within the core system, the model also considers the absence of each channel function conditions (Fig. 1d). Previous studies revealed potential interactions between CNGC-downstream factor (DF) and ClC^31,32^ and between PKG-DF and NaC^33,34^ (Methods). Therefore, our model considers four possible unknown molecular interactions within the core system (Fig. 1d, blue dashed arrows in the gray box), which were modeled by deterministic equations (see details in Methods). As shown in Fig. 1b, the growth cone MPTSs are relatively diverse despite the induction by an identical cGMP stimulation. This is likely due mainly to the difference in intrinsic properties of each growth cone. Therefore, the model also considers the peripheral parameter set, $\varphi$ (Fig. 1d), which is modeled by diffusion process and the well-established Hodgkin and Huxley quantitative mathematical model of MP regulation^35^, to characterize cell-to-cell variability, such as growth cone size (from 5 μm up to 10 μm)^36,37^ and shape that affect the intracellular cGMP diffusion rate, as well as growth cone dynamic behavior, i.e., exocytosis and endocytosis that modulate ion channel densities^38^.

### System identification based on a Bayesian framework

**Figure 2:**
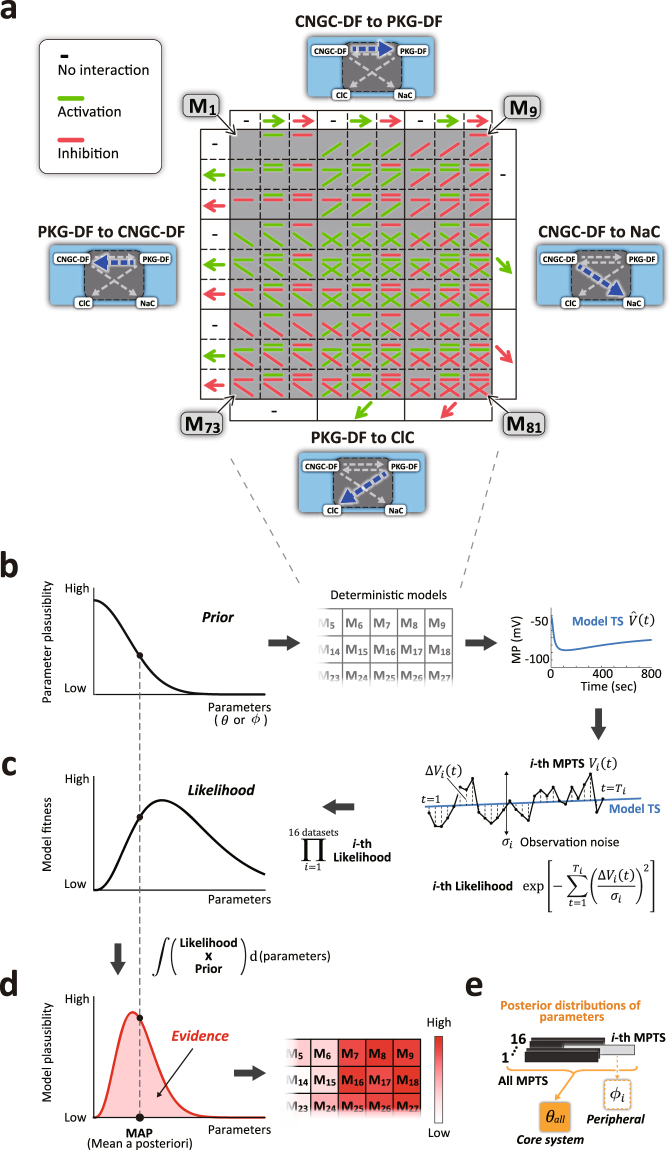
Model candidates and Bayesian system identification procedure. (**a**) The matrix shows the possible interactions in the gray region in Fig. 1d; pathways from CNGC-DF to PKG-DF, CNGC-DF to NaC, PKG-DF to ClC, and PKG-DF to CNGC-DF in the clockwise direction. Each of four pathways has three potential signals, i.e. activation (green), inhibition (red), or no interaction, as modeled by the core system parameters ($\theta$). Thus, there are ${3}^{4}=81$ model candidates labeled as M_1_ to M_81_. (**b**) Schematic illustration of prior distributions of model parameters that models the core system uncertainty and cell-dependent variability, expressing that extremely large values are biologically implausible and result in overfitting to the data (left; left-truncated Gaussians). Parameter values are applied to each of the models listed in **a** to generate model time series (TS) ($\hat{V}(t)$; blue line in the right). (**c**) Schematic illustration of model fitness, which depends on the parameters (left). The model fitness is the product of all the model likelihood (10 μM 8-Br-cGMP; control/DNDS/STX; n = 16), and the *i*-th likelihood with the parameter values in **b** was computed as the product of Gaussian probability density functions of the *i*-th MPTS (black; $V(t)$) for the entire time points ($t=1,\cdots ,{T}_{i}$) with the mean $\hat{V}(t)$ (blue) and the s.d. ${\sigma }_{i}$ (right). (**d**) Illustrates the model plausibility (left; Bayesian evidence), which evaluates both model fitness and parameter plausibility. The model plausibility was computed by integrating the product of the model fitness and the parameter plausibility over the entire ranges of all the parameters (red area in the left). The logarithmic evidences for all the 81 models were displayed in the matrix corresponding to that in **a** with red-white color (right; higher evidence is colored by red). (**e**) The core system parameters were estimated from all the datasets (${\theta }_{all}$; n = 16) and cell-dependent peripheral parameters of the *i*-th cell (${\varphi }_{i}$; n = 1) were from only the *i*-th cell’s MPTS, as posterior probability distributions. When we use representative specific parameter values instead of distributions during validation steps, we selected mean a posteriori (MAP) values (black circle in **d**) and denote the parameter set with superscript “MAP” like ${\theta }_{all}^{MAP}$.

In the mathematical model, we considered three potential interactions between one molecule and another within the core system: activation, inhibition, and no interaction. Considering the four possible unknown molecular interactions within the core system (Fig. 1d), a total of 81 (${3}^{4}=81$) possible models arise, from the simplest model, M_1_ (Model 1) with no interaction to the most complex model, M_81_ with four possible interactions (M_1_ to M_81_; Fig. 2a). We examined the model fitness of each of these 81 deterministic candidate models. Briefly, we computed the model MPTS using the core system and peripheral parameters ($\theta$ and $\varphi$) constrained by prior probability distributions (Fig. 2b). Due to our configuration of model candidates, complex models have more capability to fit the given dataset than the simple ones that are enclosed by the complex ones (e.g. M_81_ encloses M_1_). To extract a sufficient condition of signaling pathways, it is necessary to give room for complex models to approximate simple ones by reducing the values of parameters that are not common between the complex and the simple ones. We designed the priors as left-truncated normal distributions for most of the model parameters to satisfy this requirement. If using a log-normal distribution or a Gamma distribution whose shape parameter is greater than one, the probability density of parameter value being zero is zero. In contrast, a left-truncated normal distribution where the peak probability density becomes zero is useful because complex model can cut off unnecessary pathways by setting unnecessary parameters to zero. Thus we introduced the left-truncated Gaussians for the priors of the most model parameters (Supplementary Table 1). We determined the prior s.d. values based on the Monte Carlo simulation of cGMP diffusion (Supplementary Fig. 4), the number of binding sites of cGMP to CNGC and PKG, and the range and time scale of MP shift of experimental measurements. We then compared the error between the single experimental MPTS and model MPTS as the likelihood function. Due to the presence of cell-dependent noise in the experimental MPTS (Fig. 1b,c; Supplementary Fig. 1), we standardized the error by each noise level (Fig. 2c, right). We estimated the noise level ($\,{\sigma }_{i}$) from the errors between the sampled MPTS (green dots in Fig. 1c) and the smoothed time average of the same MPTS (red line in Supplementary Fig. 1; see Methods). This process was iterated for total given data points of all the MPTSs and multiplied all the likelihoods to obtain the total likelihood as model fitness for all the data sets. The model fitness, which depends on the parameter values (Fig. 2c, left), provides only the similarity between the experimental and the model MPTSs and ignores the parameter plausibility. Thus, the model fitness alone is insufficient to select a plausible model, as the parameters are likely to take unreasonable values when the model overfits the data set.

Therefore, we further computed the model plausibility by incorporating the parameter plausibility using a Bayesian framework that avoids the parameters from overfitting the data set. The model plausibility is then expressed as the product of the likelihood as model fitness and the prior as parameter plausibility. To take into account the model plausibility for the entire parameter range, we calculated the integral of the product, the Bayesian evidence^29,30^ (see Methods; Fig. 2d, left) for each of the total 81 deterministic models (Fig. 2d, right), by a Monte Carlo simulation (Supplementary Methods). We took the logarithm of the evidence (log-evidence) as index of model plausibility; the larger the log-evidence, the more plausible the model. For parameter estimation, we computed the core system parameters that were estimated from all the experimental MPTS data sets (total n = 16: 10 μM 8-Br-cGMP, control, n = 7; with DNDS blocking ClC, n = 5; with STX blocking NaC, n = 4) expressed as ${\theta }_{all}$ (Fig. 2e). The peripheral parameters (expressed as $\,{\varphi }_{i}$), which are cell-dependent, were only estimated from the *i*-th MPTS from the total MPTS data sets for computing the model fitness (Fig. 2e). As we aim to identify the molecular signaling network within the core system from the 10 μM 8-Br-cGMP-induced MPTS data set, we utilized the core system parameters that were estimated from the total data sets for the validation, specificity, and predictability tests.

### Identification and validation of the core system

**Figure 3:**
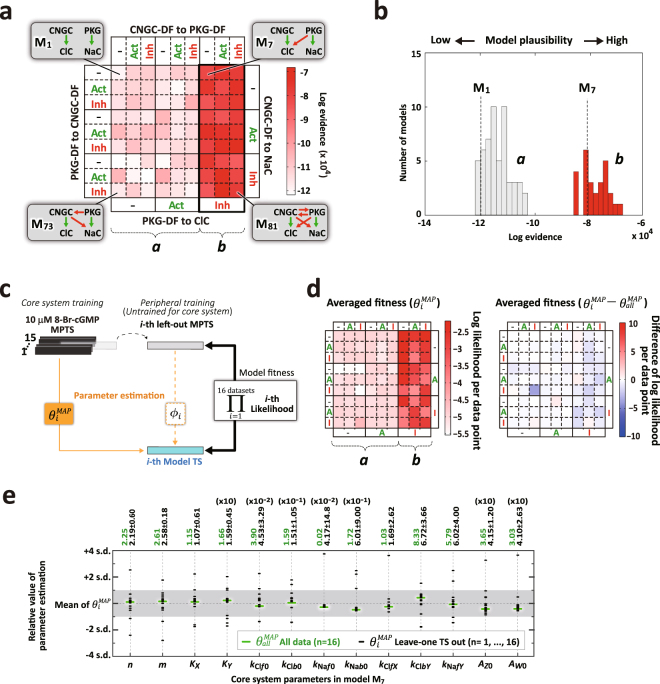
Most plausible model and the generality test by leave-one-out cross validation. (**a**) Computed model evidences (logarithm evidences; Fig. 2d) are expressed as a matrix representation following Fig. 2a (10 μM 8-Br-cGMP; control/DNDS/STX; n = 16). A model with large log evidence (toward greater red) indicates greater plausibility than that with smaller log evidence (toward white). Models in left six columns are labeled as model group *a*; those in right three columns are labeled as model group *b*. Models indicated by M_1_ and M_7_ (insets) contain, respectively, the fewest and common interactions in groups *a* and *b*, representing minimal models within the respective model groups. (**b**) Histograms of log evidence in the model groups *a* and *b*, as shown in **a**. Vertical dashed lines indicate the log evidence of the two representative models, M_1_ and M_7_. (**c**) Schematic procedure of the generality test of the core system parameter estimations by applying the leave-one-out (LOO) data selection to the models. Black and gray horizontal bars represent experimental MPTS. When the $i$-th MPTS is left out from the 10 μM 8-Br-cGMP datasets (n = 16), it is used to estimate peripheral parameters (${\varphi }_{i}$) and validate model fitness (product of all the likelihood in white box), whereas the remaining MPTS (n = 15) are completely separated from the left-out MPTS to estimate the MAP core system parameters (${\theta }_{i}^{MAP}$). Repeating LOO for all the datasets, 16 system parameter sets are obtained (${\theta }_{i}^{MAP};i=1,\,\cdots ,\,16$). The model fitness to the left-out MPTS was taken the logarithm and averaged over all the data points. (**d**) Matrix representation of the averaged model fitness by LOO-derived parameter (${\theta }_{i}^{MAP}$) (left) and the difference between the fitness and that computed from all the dataset (${\theta }_{all}^{MAP}$) (right). (**e**) Distributions of the estimated MAP core system parameter values given by LOO in **a** (${\theta }_{i}^{MAP}$; black point) compared with those by all the datasets (${\theta }_{all}^{MAP}$; green circle; n = 16). The parameters exhibited in the horizontal axis and their values are represented in the vertical axis with relative representation with respect to the mean (actual values are listed in upper axis with the corresponding color). Most of the estimations are within the mean ± s.d. (gray region).

As shown in Fig. 3a, the evidence computed from the experimental MPTS data sets (n = 16; see Methods) for each deterministic models revealed two distinct groups: group *a* with smaller evidences (light pink region) and group *b* with larger evidences (red region) (Fig. 3a). The difference of the evidences between the models within each group is much smaller than between the two groups (Fig. 3b), suggesting that the molecular pathway commonly present in the group *b* models may play a significant role. Noticeably, M_7_, which represents the simplest molecular interactions present within group *b*, reveals that PKG-mediated ClC inhibition is involved in cGMP signaling during MP shifts.

To evaluate how stable the core system parameters were estimated, we examined the generality of the estimated core system parameters by performing leave-one-out (LOO) cross validation (LOO-CV)^39^. We first separated the 16 data sets into two subsets: one with 15 data sets to train the core system parameters (${\theta }_{i}^{MAP}$; except for the *i*-th MPTS) and the other with only one data set (left-out MPTS) to train the peripheral parameters (${\varphi }_{i}$; the *i*-th MPTS). The left-out MPTS was used for computing the likelihood as an untrained data, as it was not incorporated into the core system parameters. We iterated the training and computing procedure 16 times for the total samples ($i=1,\cdots ,16$) and multiplied 16 likelihoods to obtain the model fitness (Fig. 3c; see Method). If the core system parameter estimation is stable, the matrix representation of model fitness to untrained data sets should be similar to that of the trained data sets and the core system parameters in M_7_ should not be significantly altered by LOO-CV. The average of the model fitness values (averaged fitness with $\,{\theta }_{i}^{MAP}$), which are expressed in log likelihood per data point (green dots in Fig. 1c), appears small in group *a* and large in group *b* (Fig. 3d, left panel). This average fitness shows insignificant differences between LOO data sets (${\theta }_{i}^{MAP}$) and all data sets (${\theta }_{all}^{MAP}$) (Fig. 3d, right panel), suggesting that the untrained MPTS (left-out MPTS) can be reproduced by the original models with the parameters estimated from the 15 separated MPTSs. In addition, the maximum log likelihood evaluation for the training with LOO data set (Supplementary Fig. 8) showed that the models are clustered into same two groups as those by the evidence (Fig. 3a,b). Although these are only maximum likelihoods, considering Supplementary Fig. 7, these results suggest that model group *b* is stably superior to group *a* independent of data set combination within LOO-CV. Furthermore, for the core system parameter values, the computation that resulted from performing LOO-CV shows that each of the mean a posteriori (MAP) parameters in M_7_ (${\theta }_{i}^{MAP};$ $i=1,\cdots ,16$) is close to the original MAP parameters estimated from the complete data set (${\theta }_{all}^{MAP}$) (Fig. 3e; within the LOO mean ± s.d.). These results verify that our identification method, which depends on the stability of the core system parameters, is not significantly affected by the data set combination of LOO-CV, indicating that the estimated core system parameters are reasonably stable.

### Specificity of trained membrane potential time series

**Figure 4:**
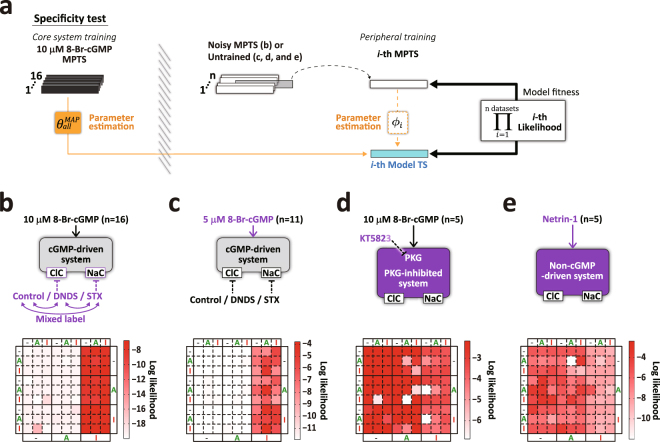
Specificity of identified core system to 8-Br-cGMP-induced MPTS. (**a**) Schematic procedure of model specificity test using MPTSs in different conditions using MAP core system parameters estimated from all the 10 μM 8-Br-cGMP datasets (${\theta }_{all}^{MAP}$; n = 16; black horizontal bars in the left side of the shaded band). The model likelihood to the $i$-th MPTS from other testing datasets (white bars) was computed while computing the cell-dependent parameters, ${\varphi }_{i}$, and by repeating this the model fitness to all the *n* datasets were obtained. Testing datasets are the datasets untrained by the core system (5 μM 8-Br-cGMP-induced MPTS, PKG-inhibited condition (KT5823), and netrin-1-induced MPTS), those with noise (10 μM 8-Br-cGMP-induced MPTS with randomly labeled channel inhibitor). (**b** and **c**) Matrix representations of model fitness by the datasets derived by the control core system; 10 μM 8-Br-cGMP-induced MPTS with mixed labels of channel conditions (control, DNDS, STX) (**b**; n = 16) by randomly selecting channel blocker conditions in the model (see Supplementary Methods), and 5 μM 8-Br-cGMP-induced MPTS (**c**; total n = 11; control: n = 2; with DNDS blocking ClC: n = 5; with STX blocking NaC: n = 4). (**d** and **e**) Same as **b** and **c**, but the datasets derived by the different core systems; 10 μM 8-Br-cGMP-induced MPTS under the PKG-inhibited condition (KT5823) (**d**), and netrin-1-induced MPTS (**e**).

Next, we tested the specificity of the identified model to the given trained data sets – 10 μM 8-Br-cGMP-induced MPTS (Fig. 1b). To test the degree of specificity, we introduced untrained data sets of MPTSs induced under different experimental conditions. We then compared their model fitness with that derived from the original trained data sets (Fig. 3a) by computing log likelihoods per data point using the core system parameters estimated from the experimental 10 μM 8-Br-cGMP MPTSs (${\theta }_{all}^{MAP}$; Fig. 4a). First, we introduced the mixed labeled channel condition models (control/STX/DNDS to random labels) (Fig. 4b). As each MPTS is the sum of ClC and NaC components, the pattern of the model fitness matrix is not expected to be significantly altered by mixing the label of the data sets. As shown in Fig. 4b, the model fitness matrix clearly shows separation of group *a* and *b* as in Fig. 3a. When we introduced the MPTS induced by a different concentration of stimulant – 5 μM, instead of 10 μM 8-Br-cGMP, the model fitness matrix shows a similar pattern of model groups, as in Fig. 3a (Fig. 4c). This might indicate that core system parameters have a small dependency on the stimulus intensity between 5 μM and 10 μM concentration of 8-Br-cGMP.

In contrast, when we introduced the MPTS induced by 10 μM 8-Br-cGMP in the presence of KT5823 (n = 5), a PKG activity inhibitor (Supplementary Fig. 3a) that abolishes the PKG activation in the core system, the model fitness matrix shows the total absence of the distinct segregation between the model group *a* and *b* (Fig. 4d). This supports that PKG activation is required for the pattern of model groups observed in Fig. 3a, at least partially. Lastly, we introduced the MPTS induced by netrin-1, a diffusible guidance molecule (Supplementary Fig. 3b) that regulates MP shifts through a different molecular signaling pathway^40^. As shown in Fig. 4e, the pattern of the model fitness matrix shown in Fig. 3a is totally abolished (Fig. 4e; Supplementary Methods), supporting that our models are specifically trained for cGMP signaling. Taken together, these tests indicate that our system identification method has high specificity.

### Predictability of the untrained late phase of MPTS

**Figure 5:**
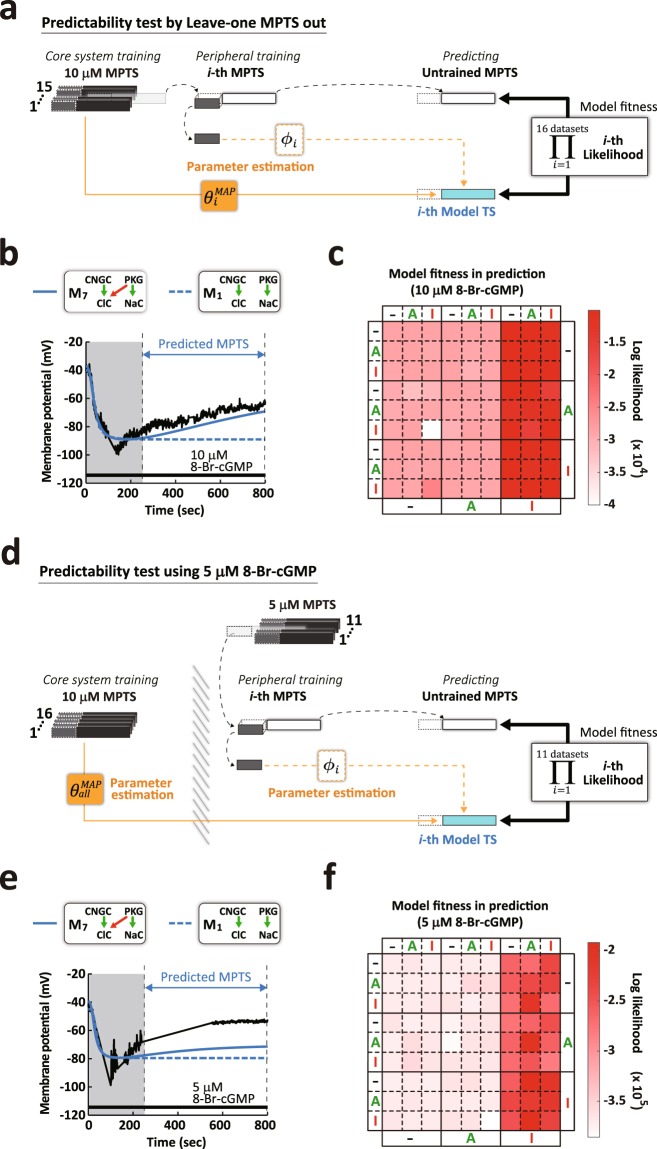
Model predictability of untrained 8-Br-cGMP-induced MPTS. (**a**) Schematic illustration of the procedure for testing model predictability for the late-phase of MPTS induced by 10 μM 8-Br-cGMP. The initial-phase (250 sec) of the left-out $i$-th MPTS was extracted to estimate the MAP peripheral parameters (${\varphi }_{i}$), and the remaining $i$-th Msas used to test the model predictability. The MAP core system parameters were estimated from the initial and late phase of the remaining MPTS (${\theta }_{i}^{MAP}$; n = 15). (**b**) Sample MPTS predicted by model M_1_ (blue dotted line) and M_7_ (blue solid line) by the prediction procedure in **a**. (**c**) Matrix representation of log-likelihood of the predicted MPTS when repeating $i=1,\cdots ,16$ and summing them. (**d**) Same as **a**, but 5 μM 8-Br-cGMP MPTS was predicted with ${\varphi }_{i}$ given the initial-phase MPTS (up to 250 sec) and ${\theta }_{all}^{MAP}$ by all the 10 μM 8-Br-cGMP MPTS. Black and white bars represent experimental MPTS. MPTS in the white regions (after 250 sec, the late-phase) were never used for the parameter estimation; these MPTSs were completely untrained data sets even for the peripheral parameters. (**e**) Sample MPTS predicted by model M_1_ (blue dotted line) and M_7_ (blue solid line) by the prediction procedure in **d**. (**f**) Matrix representation of log-likelihood of the predicted MPTS when repeating $i=1,\cdots ,11$ and summing them.

We have demonstrated that our integrated methodology for training the model parameters specifically derived from electrophysiological data sets monitored in response to cGMP stimulation in nerve growth cones has the capability to identify the core molecular system of cGMP signaling. We further determined whether the identified models in group *b* have the capability of predicting the untrained, late phase of MPTS, when the peripheral parameters were trained for the initial phase of MPTS. Although the estimation of the initial phase MPTS from the late phase is possible in principle, it is practically impossible because the late phase MPTS contains little information on the signaling pathways due to steady state of cGMP concentration. Specifically, we examined the accuracy of forecasting the late phase data points of the reference initial phase of MPTS, using a data assimilation test^41^ in which the core system, as well as the peripheral parameters for the late phase MPTS, are not incorporated. First, we set the core system parameters to the MAP values that derived from the data sets of MPTS induced by 10 μM 8-Br-cGMP, except for the *i*-th MPTS (${\theta }_{i}^{MAP}$; n = 15). The peripheral parameters were trained for the initial phase of the *i*-th left-out MPTS (${\varphi }_{i}$), which was iterated 16 times ($i=1,\,\cdots ,\,16;{\rm{n}}=16$). Subsequently, we computed the model fitness for the late phase of the MPTS (250 to 800 sec) and projected the predicted late phase of MPTS (indicated as the predicted MPTS in Fig. 5a). As shown in Fig. 5b, the representative models, M_1_ and M_7_ predicted the trajectories of the late phase MPTSs induced by 10 μM 8-Br-cGMP, demonstrating the significant superiority of M_7_ over M_1_ in its capability to predict the late phase of MPTS. Normalized root mean squared errors (RMSEs) were also computed with the time series used in Fig. 5c to examine the superiority of group *b* to group *a* by other evaluation criteria **(**Supplementary Fig. 9a). We found that RMSEs also shows that group *b* models have a high ability to predict the late phase MPTS, consistent with Fig. 5c. We further confirmed the essential requirement of the core molecular system for the predictability by replacing the 5 μM 8-Br-cGMP data sets for the peripheral parameters training. As in Fig. 5d, the core system parameters were trained for all the MPTSs of 10 μM 8-Br-cGMP data sets (${\theta }_{all}^{MAP}$; n = 16) while the peripheral parameters were trained only for the initial phase of the *i*-th left-out MPTS (${\varphi }_{i}$) induced by 5 μM 8-Br-cGMP, and the model fitness was computed similarly as in Fig. 5a ($i=1,\,\cdots ,\,11;$ Fig. 5d). RMSEs with the time series used in Fig. 5f also showed the superiority of the group *b* to the group *a* (Supplementary Fig. 9b). These tests support that the models in the group *b* have the capacity to predict the untrained late phase of MPTS given the generalized core molecular system parameters derived from the 10 μM 8-Br-cGMP MPTSs.

### Reproducibility of cGMP-dependent bidirectional MP shifts

**Figure 6:**
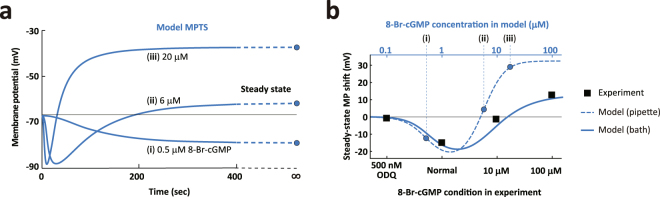
Reproducibility of cGMP-dependent MP shifts. (**a**) Model MPTS by the model M_7_ induced by different 8-Br-cGMP concentration: (i) 0.5, (ii) 6, and (iii) 20 μM with ${\theta }_{all}^{MAP}$ and ${\varphi }_{all}^{MAP}$ (Supplementary Table 1). Each MPTS reaches the steady-state at about 300 sec. We defined the model’s steady-state MP shift as the difference between the MPs at times zero and infinity, namely, $\hat{V}(t\to \infty )-\hat{V}(t=0)$, where the core system parameters $({\theta }_{all}^{MAP})$ from the 10 μM 8-Br-cGMP dataset were used. For the peripheral parameters, ${V}_{{\rm{K}}}$ is a mere constant bias in $\hat{V}(t)$ (see Supplementary Methods) and, therefore, was canceled out, ${\tau }_{S}$ was ignorable at times zero and infinity, and ${A}_{Z}$ and ${A}_{W}$ were replaced by their means in ${\varphi }_{all}^{MAP}$. (**b**) Model of 8-Br-cGMP concentration dependent MP shifts. Prediction of the cGMP-dependency of the steady-state MP shifts induced by the direct pipette stimulation (inject 8-Br-cGMP through the recording pipette; blue dashed line). Mimicking of the bath application condition of 8-Br-cGMP (blue solid line) by correcting the 8-Br-cGMP permeation process from outside to inside of the cell (Supplementary Methods). Experimental data of steady-state MP shifts under the bath application condition (black square with lower x-axis; ref.^16^) are shown for comparison. Normal condition indicates a cGMP level when Sema3A was applied in the turning assay; ODQ (1H-(1,2,4) oxadiazolo(4,3-a)quinoxalin-1-one, a soluble guanylyl cyclase inhibitor) condition reduces growth-cone cGMP levels.

Upon the injection of cGMP stimulant through the recording pipette into the nerve growth cone, the resting membrane potential (from about $-80$ to $-70$ mV) becomes hyperpolarization^16^. As the intracellular cGMP increases, the hyperpolarization slowly converts to depolarization, causing a bidirectional MP shift^16^. We simulated these bidirectional MP shifts, which show temporal hyperpolarization in the initial phase of the stimulation that converts to depolarization and eventually reaches the steady state (after 10 min; Fig. 6a). At the steady state, MPs maintain almost constant values, which have bidirectional dependency on the level of 8-Br-cGMP stimulation^16^. We examined whether the core molecular system containing the PKG inhibition of ClC that was highlighted in M_7_ is also required during the induction of cGMP-dependent bidirectional MP shifts. We computed the M_7_ with ${\theta }_{all}^{MAP}$ estimated from 10 μM 8-Br-cGMP (n = 16) at different concentrations of cGMP. Our model simulation shows: (i) a gradual hyperpolarization stimulated by a low 8-Br-cGMP (0.5 μM) concentration, (ii) a sharp hyperpolarization that gradually recovered to the resting MP level stimulated by a moderate 8-Br-cGMP (6 μM) concentration, and (iii) a sharp hyperpolarization that converted to depolarization stimulated by a high concentration of 8-Br-cGMP (20 μM). We then compared the steady state MP of the model (blue circles in Fig. 6a) with the experimental data (Fig. 5e in ref.^16^). The simulated cGMP concentration-dependent MP shifts show a bidirectional MP shift (Fig. 6b, a blue dotted line). Although our model simulation showed bidirectional MP shifts, the magnitude of depolarization was much greater and the occurrence of depolarization appeared at much lower 8-Br-cGMP concentration than that which was observed experimentally. This difference may result from the difference between the modeled direct stimulation by 8-Br-cGMP through the recording pipette compared to the bath application of 8-Br-cGMP in the experiment^16^. When we introduced a simple Hill-like model of membrane permeation of 8-Br-cGMP into the model to mimic the bath-application of 8-Br-cGMP (Supplementary Fig. 6; Supplementary Methods), indeed, M_7_ (blue solid line in Fig. 6b) was able to reproduce the bidirectional MP shifts that were much closer to those observed experimentally^16^ (black squares in Fig. 6b).

## Discussion

We present a computational analysis that reveals an essential molecular signaling pathway within the core system of MP shifts recorded from a growth cone in response to an external stimulus that directs growth cone turning. We show a novel integrated reverse-engineering of the system comprised of different physical quantities and Bayesian framework methods that accommodate the large cell-to-cell variability and small number of data sets that otherwise hinder the biophysical modeling. By implementing the Bayesian framework, we specifically show the optimization of peripheral parameters for individual cells that overcomes the cell-to-cell variability, and the core system parameters for all given data sets to extract an unknown molecular pathway. Thus, our parameter categorization is especially useful for extracting common molecular pathways involved in electrophysiological responses in a cell.

The model plausibility expressed by Bayesian evidence^29,30^ evaluates the overall possible parameter ranges, unlike the Akaike information criterion^42^ (AIC; Supplementary Fig. 7a) and the Bayesian information criterion^43^ (BIC; Supplementary Fig. 7b), and the maximum log likelihood (Supplementary Fig. 7c). AIC and BIC did not show clearly divided criteria values as shown in Fig. 3a,b. These model selections successfully function when the number of data is large enough. The maximum log likelihood clearly divided likelihood values as shown in Fig. 3a,b. However, the evaluation of the likelihood function is performed at one specific parameter set (maximum likelihood parameters) and hence it is unclear whether high likelihood is also given around this parameter set. Thus, it allows reasonable model selection even when a small number of data sets are given. Normal approaches to deduce a signaling pathway, such as a biophysical modeling, although the model accuracy may be higher, demands a large number of data sets to estimate not only the specific values of the core system parameters, but also the distributions of the peripheral parameters. Collecting a large number of biological data sets is laborious and time-consuming, and data sets such as the MPTSs in this work are difficult to measure. Our computational methodology demonstrates the feasibility of extracting a hidden core molecular system from a small number of data sets and with a large cell-to-cell variability. Thus, our computational analysis, at the least, has the capacity of ranking the possibility of the biomolecular system, which significantly reduces the laborious task and extensive time required by conventional experimentation.

Our computational analyses reveal that PKG-mediated ClC inhibition is an essential pathway that acts in concert with CNGC-mediated ClC activation and PKG-mediated NaC activation, as demonstrated in model M_7_. In support of our model prediction, biochemical studies have shown that PKG, indeed, is a regulator of the Mitogen-activated Protein Kinase (MAPK), such as ERK and p38^44^, both of which inhibit calcium-dependent ClC^45^. Regarding the parameter values, the bidirectional cGMP-dependent MP shifts (Fig. 6b) showed that CNGC-mediated ClC activation is due to a high-affinity of cGMP (${K}_{X}=1.15$ in Fig. 3e), whereas PKG-mediated NaC activation is due to a low-affinity of cGMP (${K}_{Y}=16.61$ in Fig. 3e). The bidirectional phenomenon based on a difference in the dissociation constants of positive and negative regulators has been shown in the molecular system of synaptic plasticity, for example, in the phosphorylation of α-amino-3-hydroxy-5-methyl-4-isoxazolepropionic acid (AMPA) receptors that occurs in competition between the kinase and phosphatase^46^. Our study suggests a novel regulatory mechanism of the bidirectional cGMP-dependent MP shifts in growth cone guidance in which PKG not only facilitates membrane depolarization^16^, but also simultaneously inhibits hyperpolarization. Thus, PKG-mediated ClC inhibition could facilitate the dynamic range of MP shifts by opposing a stimulatory input at a small range of 0.1–10 μM cGMP concentration that contributes to the overall dynamic range of the growth cone turning response.

## Methods

### Data preprocessing

Analyzed experimental data of membrane potential (MP) were recorded from growth cones of cultured *Xenopus* spinal neurons as described previously^16^. The recorded MP time series (MPTS) contain significant noises and we considered three types of noises (Supplementary Fig. 1): 1. spike-like system noise, 2. step-like temporal change that likely due to experimental artifacts, and 3. small observation noises, like thermal fluctuation. In the analysis, we used the sampled MPTS of 300–900 data points (one sec interval; green dots in Fig. 1c) from the raw data (>10000 data points; black line in Fig. 1c) that removed most of the spike-like system noise. The step-like artifacts were complemented with a straight line connecting the onset point and the end point of the temporal change (one sec interval; blue points in Supplementary Fig. 1). On the other hand, we disregarded the small observation noise, as it is too small to remove. Instead, we used scaling model fitness (difference between the data point and the mathematical model). We modeled the small observation noise for each MPTS by a white Gaussian with the s.d. (${\sigma }_{i}$ in Fig. 2c), which was estimated from the difference between the sampled MPTS data points and the smoothed data points (red line in Supplementary Fig. 1. Such noise model was applied for standardizing error between observed and model data.

### MP dataset

We have previously recorded MPTS under following stimulation and culture conditions^16^:

1. 10 μM 8-Br-cGMP in the recording pipette, as a stimulant (control, n = 7; Fig. 1b), and in the presence of either chloride channel (ClC) blocker (DNDS; n = 5; Supplementary Fig. 2a), or sodium channel (NaC) blocker (STX; n = 4; Supplementary Fig. 2b) in the culture bath.
2. 5 μM 8-Br-cGMP in the recording pipette, as a stimulant (control, n = 2; Supplementary Fig. 2c), and in the presence of either chloride channel (ClC) blocker (DNDS; n = 5; Supplementary Fig. 2d), or sodium channel (NaC) blocker (STX; n = 4; Supplementary Fig. 2e) in the culture bath.
3. 10 μM 8-Br-cGMP in the recording pipette as a stimulant in the presence of PKG inhibitor (KT5823; n = 5; Supplementary Fig. 3a).
4. 5 μM netrin-1, as a stimulant (control, n = 5; Supplementary Fig. 3b).

The 10 μM 8-Br-cGMP-stimulated datasets that include control, DNDS, and STX, were used for system identification, as they provide the largest number of datasets. The remaining datasets were used for the validation test.

### Molecular signalling pathways

Previously, we have shown^16^ that during bath application of pharmacological drugs to cultured neurons in the presence of 8-Br-cGMP stimulation: 1. MP shifts to depolarization in the presence of DNDS, the ClC blocker; 2. MP shifts to hyperpolarization in the presence of STX; and 3. Application of a PKG inhibitor, KT5823, caused sustained hyperpolarization, supporting that ClCs are required for hyperpolarization; NaCs are required for depolarization; and PKG activity is required for depolarization (Supplementary Fig. 5). It has also been demonstrated that the cGMP-induced hyperpolarization is, in part, due to the activation of CNGCs via cGMP directly activating the channels^14,47^, which ultimately activates the hyperpolarizing Cl channels (ClCs)^31,32^. Likewise, the cGMP-activated PKG, is known to be a regulator of the Mitogen-activated Protein Kinase (MAPK) such as p38^44^, which activates a TTX-resistant sodium channel, Nav1.8^33,34^. Thus, we incorporated these known pathways of NaC and ClC activation by PKG and CNGC, respectively, into our model (Fig. 1d,e).

### Bayesian formulation for the parameter estimation

Because the parameters of growth cone volumes and ion channel densities affect the MP shifts, we applied a Bayesian framework. The model parameters were categorized into three classes: core system and peripheral parameters, and experimentally derived parameters (Supplementary Table 1).

The core system parameter set, which includes the biochemical reaction rates, Hill coefficients, and means of ${A}_{{\rm{Cl}}}$ and ${A}_{{\rm{Na}}}$ (${A}_{{\rm{Cl0}}}$ and ${A}_{{\rm{Na0}}}$, respectively), was estimated from the total MP data sets, as it is a common system for all neurons.

On the other hand, the peripheral parameter set, $\varphi =\{{V}_{K},\,{A}_{{\rm{Cl}}},\,{A}_{{\rm{Na}}},\,{\tau }_{S}\}$, which is highly dependent on characteristics of individual neurons (e.g., growth cone volume, channel densities) was estimated for each MP time series. The experimental condition parameter set, $c=\{{\eta }_{{\rm{Cl}}},\,{\eta }_{{\rm{Na}}},\,{S}_{max}\}$, represents the experimentally derived parameters, such as pharmacological condition and applied 8-Br-cGMP concentration.

By the Bayesian approach, the model parameters were estimated from the experimental data under constraints given by prior distributions (mostly left-truncated Gaussians; see Supplementary Table 1). The Bayes’ theorem delivers the posterior distribution of the model parameters as,

1 $$p(\varphi ,\theta |V,M)=\frac{p(V|\varphi ,\theta )p(\varphi ,\theta |M)}{E(V,M)}$$

2 $$E(V,M)={\iint }^{}p(V|\varphi ,\theta ,M)p(\varphi ,\theta |M)d\varphi d\theta .$$

Here, the experimental condition parameter set, $c$, is omitted for easy visibility. The evidence, $E(V,M)$, is the model evaluation criteria (Fig. 2d) determined by the MP dataset, $V$, and the model, $M$. The parameters of the prior distributions, $p(\varphi ,\theta |M)$ (Fig. 2b), were listed in Supplementary Table 1. The likelihood, which represents the fitness level to the given dataset, is given by the product of the Gaussians (Fig. 2c),

3 $$\begin{array}{rcl}p(V|\varphi ,\theta ) & = & \prod _{i=1}^{I}p({V}_{i}|{\varphi }_{i},\theta )=\prod _{i=1}^{I}\prod _{t=1}^{{T}_{i}}\frac{1}{\sqrt{2\pi }{\sigma }_{i}}\exp (-\frac{{\rm{\Delta }}{V}_{i}^{2}(t)}{2{\sigma }_{i}^{2}})\\  & = & {(\frac{1}{\sqrt{2\pi }{\sigma }_{i}})}^{I}\exp \{-\frac{1}{2}\sum _{i=1}^{I}\sum _{t=1}^{{T}_{i}}{(\frac{{\rm{\Delta }}{V}_{i}(t)}{{\sigma }_{i}})}^{2}\},\,\end{array}$$

where ${\rm{\Delta }}{V}_{i}(t)$ is defined as the MP difference at the time $t$ (Fig. 2c), ${\rm{\Delta }}{V}_{i}(t)={V}_{i}(t)-\hat{V}(t|{\varphi }_{i},\theta )$, indicating the error between the observed MP, ${V}_{i}(t)$, and the model MP, $\hat{V}(t|{\varphi }_{i},\theta )$ (Eq. (S15) in Supplementary Methods), where $i$ is the data index ($I=16$ and ${T}_{i}=300 \sim 900$). The s.d. of the $i$-th MP time series, ${\sigma }_{i}$, was the size of observation noise, which was computed during the data preprocessing. Model fitness expressed as likelihood is defined in Fig. 2c, and is obtained by taking the natural logarithm of Eq. (3), ignoring constant parameters. The practical calculation of the evidence was performed by Markov Chain Monte Carlo (MCMC) simulation (Supplementary Methods).

### Computation software and time

All computations were performed with Matlab (MathWorks) and its parallel computing toolbox. We used up to 1,000 core CPUs for Monte Carlo simulation. The calculation of a single evidence took about a day per model (Fig. 3a), and that of a single likelihood took from a half day to a day per validation (Figs 3d, 4b–e, and 5c,f).

### Data availability

All data and code used to perform analyses reported herein are available from the corresponding author at reasonable request.

## Electronic supplementary material

Supplementary Information
