# Supplementary material for: Computational Methods for Estimating Molecular System from Membrane Potential Recordings in Nerve Growth Cone
Source: Sci Rep. 2018 Mar 14;8:4559. doi: 10.1038/s41598-018-22506-3 (PMC5852145; doi:10.1038/s41598-018-22506-3)
Supplement: Supplementary file 1 — Supplementary Information [file 41598_2018_22506_MOESM1_ESM.pdf]

Supplementary information for

# Computational Methods for Estimating Molecular System from Membrane Potential Recordings in Nerve Growth Cone

Tatsuya Yamada, Makoto Nishiyama, Shigeyuki Oba, Henri Claver Jimbo,
Kazushi Ikeda, Shin Ishii, Kyonsoo Hong*, Yuichi Sakumura*

**This PDF file includes:**

| | Title |
|---|---|
| Supplementary Methods | |
| Supplementary Figure 1 | Preprocessing of membrane potential time series |
| Supplementary Figure 2 | Membrane potential time series (MPTS) recorded from growth cone of *Xenopus* spinal neuron |
| Supplementary Figure 3 | MPTS induced by the different system |
| Supplementary Figure 4 | Computation of the 8-Br-cGMP diffusion rate |
| Supplementary Figure 5 | Pharmacological application inducing MP shift |
| Supplementary Figure 6 | Model of extracellular 8-Br-cGMP permeating into cell cytoplasm via plasma membrane |
| Supplementary Figure 7 | Comparison of model selection criteria |
| Supplementary Figure 8 | Maximum log likelihood of each core system training step during leave one out (LOO) procedure |
| Supplementary Figure 9 | Normalized RMSE of model predictability of untrained 8-Br-cGMP-induced MPTS |
| Supplementary Table 1 | Summary of prior distributions of model parameters |

## Supplementary Methods

**Time constant of 8-Br-cGMP diffusion.**
In the experiment, the recording pipette filled with solution of 8-Br-cGMP was attached near the center of the growth cone palm, from which cGMP analogues (8-Br-cGMP) diffused over the entire growth cone. The time series of the 8-Br-cGMP concentration in the model growth cone, $S$, was approximated as the simple exponential function of time $t$,

$$S = S_{\max}(1 - e^{-t/\tau_S}), \qquad \text{(S1)}$$

where $S_{\max}$ is the 8-Br-cGMP concentration in the pipette and $\tau_S$ is the diffusion time constant. The diffusion time constant is a cell-dependent peripheral parameter, as it depends on the volume of a growth cone.

To express cell variability in the time constant, $\tau_S$, it was modeled as the probability distribution estimated from the numerical calculation of a model growth cone consisting of three shell compartments (**Supplementary Fig. 4a**). The inner two shell compartments have 1 μm thickness and the outer shell compartment has a variable thickness of 1 to 2 μm modified by Gaussian noise $\varepsilon \sim N(0, 1^2)$. In addition, we added one pipette compartment, indexed by 0, in which the cGMP concentration is the maximum with a fixed concentration. The 8-Br-cGMP concentration in the $n$-th compartment, $S_n$ (μM), should obey the Fick's law,

$$\frac{d}{dt}S_n = D\frac{A_{n-1,n}}{d_{n-1,n}V_n}(S_{n-1} - S_n) + D\frac{A_{n,n+1}}{d_{n,n+1}V_n}(S_{n+1} - S_n), \qquad \text{(S2)}$$

where $D$, $A_{n-1,n}$, $d_{n-1,n}$, and $V_n$ are the diffusion coefficient of 8-Br-cGMP (1 μm$^2$/s)[1], the surface area between the $(n-1)$-th and the $n$-th compartments, the distance between the compartment midpoints, and the volume of the $n$-th compartment, respectively. We solved Eq. (S2) numerically with the boundary condition, $S_0 = S_{\max}$, and confirmed that the time course in the third compartment (the outer shell) can be approximated by the exponential function in Eq. (S1) (**Supplementary Fig. 4b**). Monte Carlo simulation with various growth cone sizes gave the distribution of the time constant, $\tau_S$, whose mean was 40 sec.

**Molecular signalling pathways.**
Previously, we have shown[2] that during bath application of pharmacological drugs to cultured neurons in the presence of 8-Br-cGMP stimulation: 1. MP shifts to depolarization in the presence of DNDS, the ClC blocker; 2. MP shifts to hyperpolarization in the presence of STX; and 3. Application of a PKG inhibitor, KT5823, caused sustained hyperpolarization, supporting that ClCs are required for hyperpolarization; NaCs are required for depolarization; and PKG activity is required for depolarization (**Supplementary Fig. 5**). It has also been demonstrated that the cGMP-induced hyperpolarization is, in part, due to the activation of CNGCs via cGMP directly activating the channels[3,4], which ultimately activates the hyperpolarizing Cl channels (ClCs)[5,6]. Likewise, the cGMP-activated PKG, is known to be a regulator of the Mitogen-activated Protein Kinase (MAPK) such as p38[7], which activates a TTX-resistant sodium channel, Nav1.8[8,9]. Thus, we incorporated these known pathways of NaC and ClC activation by PKG and CNGC, respectively, into our model (**Fig. 1d** and **e**).

**Modeling of CNGC- and PKG-downstream pathways upon the 8-Br-cGMP stimulation.**
The model considers the activation of CNGCs and/or PKGs upon binding to 8-Br-cGMP (**Fig. 1d** and **e**). The activity levels of CNGC- and PKG-downstream factors (DFs), expressed as $X$ and $Y$, respectively increase, as the level of 8-Br-cGMP concentration increases, the DFs' activities reach their steady-state (or saturation) levels on a time scale of the minute. We applied quasi-steady state approximation to express $X$ and $Y$ as Hill-type functions of $S$,

$$X = \frac{S^n}{K_X + S^n}, \quad K_X = \frac{k_{Xb}}{k_{Xf}} \qquad \text{(S3)}$$

$$Y = \frac{S^m}{K_Y + S^m}, \quad K_Y = \frac{k_{Yb}}{k_{Yf}}, \qquad \text{(S4)}$$

where the Hill coefficients, $n$ and $m$, are effectively triggeing the downstream processes. The activity levels, $X$ and $Y$, are normalized in the range [0, 1], and the actual activity levels are expressed by multiplying their maximum values.

The dissociation constants, $K_X$ and $K_Y$, are defined by the ratio of backward rates ($k_{Xb}$ and $k_{Yb}$) to forward rates ($k_{Xf}$ and $k_{Yf}$) of the reactions between the levels of intracellular 8-Br-cGMP concentration and the DFs' activities. Applying the linear approximation to forward and backward reaction rates, the effective dissociation

constants are written as,

$$K_X = K_X(Y) = \frac{k_{Xb0} + k_{XbY}Y}{k_{Xf0} + k_{XfY}Y} \qquad \text{(S5)}$$

$$K_Y = K_Y(X) = \frac{k_{Yb0} + k_{YbX}X}{k_{Yf0} + k_{YfX}X}\ , \qquad \text{(S6)}$$

where the denominator and numerator represent the forward and backward reactions, respectively. The values of the eight parameters in Eqs. (S5) and (S6) are involved in the core system parameter set, $\theta$, and reflect the structure of model pathways. For instance, only $k_{XbY}$ takes zero in Eq. (S5) if PKG-DF accelerates the forward reaction of CNGC-DF, and both of $k_{XfY}$ and $k_{XbY}$ are zero if PKG-DF has no pathway to CNGC-DF. Therefore, each of these equations can represent three possible interactions: activation, inhibition, and no interaction between one molecule and another. Thus the total of nine possible interactions can be considered ($3 \times 3$ possible interactions in horizontal pathways in **Fig. 2a**). In the estimation of the parameters, we divided Eqs. (S5) and (S6) by $k_{Xf0}$ and $k_{Yf0}$, respectively, to reduce the effective number of parameters to be estimated (see **Supplementary Table 1**).

**Modeling ion channel activities.**

As MP shifts depend on channel densities[10], our deterministic model considers the regulation of both ClCs and NaCs densities by CNGC- and PKG-DFs. The dynamics of the channel densities are defined by the changes in ratios of channel densities, which are expressed as $D_{\mathrm{Cl}}$ and $D_{\mathrm{Na}}$ for ClCs and NaCs, respectively, and their constant values indicate no regulation, e.g., in the absence of stimulation; variable values indicate the presence of regulation, e.g., in the presence of the cGMP stimulation. Since the regulation of $D_{\mathrm{Cl}}$ and $D_{\mathrm{Na}}$ depend on $X$ and/or $Y$, the core system $\theta$ limits the rate of the signal processing in the model (minute time scale). Thus, the dynamics of the biochemical reactions is described by ODEs,

$$\frac{dD_{\mathrm{Cl}}}{dt} = k_{\mathrm{Cl}f}(1 - D_{\mathrm{Cl}}) - k_{\mathrm{Cl}b}D_{\mathrm{Cl}} \qquad \text{(S7)}$$

$$\frac{dD_{\mathrm{Na}}}{dt} = k_{\mathrm{Na}f}(1 - D_{\mathrm{Na}}) - k_{\mathrm{Na}b}D_{\mathrm{Na}}\ , \qquad \text{(S8)}$$

where $k$ with a subscript is the effective forward or backward reaction rate, which is either a constant or a function of the normalized variables, $X$ and/or $Y$. By the linear approximations, the regulations by the CNGC- and PKG-DFs, respectively, $X$ and $Y$, are described as,

$$k_{\mathrm{Cl}f} = k_{\mathrm{Cl}f}(X, Y) = k_{\mathrm{Cl}f0} + k_{\mathrm{Cl}fX}X + k_{\mathrm{Cl}fY}Y \qquad \text{(S9)}$$

$$k_{\mathrm{Cl}b} = k_{\mathrm{Cl}b}(X, Y) = k_{\mathrm{Cl}b0} + k_{\mathrm{Cl}bX}X + k_{\mathrm{Cl}bY}Y \qquad \text{(S10)}$$

$$k_{\mathrm{Na}f} = k_{\mathrm{Na}f}(X, Y) = k_{\mathrm{Na}f0} + k_{\mathrm{Na}fX}X + k_{\mathrm{Na}fY}Y \qquad \text{(S11)}$$

$$k_{\mathrm{Na}b} = k_{\mathrm{Na}b}(X, Y) = k_{\mathrm{Na}b0} + k_{\mathrm{Na}bX}X + k_{\mathrm{Na}bY}Y, \qquad \text{(S12)}$$

where the reaction rates, $k_{**0}$, are constant (independent of $X$ or $Y$) while the others are at the maximum rates contributed by $X$ or $Y$. All the parameters in Eqs. (S9)–(S12) are the part of the core system parameter set, $\theta$, and govern the core system of the model with the parameters in Eqs. (S5) and (S6).

Among these potential four interactions, the ClC activation by CNGC-DF ($k_{\mathrm{Cl}fX}$ in Eq. (S9)) and NaC activation by PKG-DF ($k_{\mathrm{Na}fY}$ in Eq. (S11)) are known pathways, while the remaining interactions are unknown (**Fig. 2a**). We excluded the simultaneous combinations of forward and backward regulations on the same effectors. Thus, we considered three possible effects: activation, inhibition, and no interaction in each regulation of NaC by CNGC-DF or ClC by PKG-DF, which gives rise to the total of nine possible interactions to be considered ($3 \times 3$ possible interactions in cross pathways in **Fig. 2a**).

In Eqs. (S9) – (S12), we assumed no feedback pathways from the channels to the CNGC- and PKG-DFs. Although feedback pathways with some conditions lead to instability of the system's state such as divergence, oscillation[11], or bistability[12], no such unstable responses were observed in the traces of MPTS (**Fig. 1b, c**, and **Supplementary Fig. 2**).

**Linear representation of membrane potential.**

The channel densities, $D_{\rm Cl}$ and $D_{\rm Na}$, regulate the MP, and the signal conversion from the ion channel densities to the MP is well established by Hodgkin and Huxley equation[10],

$$C_m \frac{dV}{dt} = -g_{\rm Na}(V - V_{\rm Na}) - g_{\rm K}(V - V_{\rm K}) - g_{\rm Cl}(V - V_{\rm Cl}) \ , \qquad \text{(S13)}$$

with the membrane capacitance, $C_m$, the membrane potential (MP), $V$, the reversal potentials, $V_{\rm Na}$, $V_{\rm K}$, and $V_{\rm Cl}$, and the channel conductances, $g_{\rm Na}$, $g_{\rm K}$, and $g_{\rm Cl}$, which depend on channel density and/or MP. The potassium conductance at the resting state has a dominant permeability ($g_{\rm K} \gg g_{\rm Cl}, g_{\rm Na}$). By setting the Eq. (S13) to zero, the MP at the steady state and resting state are approximately given by

$$V = \frac{\bar{g}_{\rm K}V_{\rm K} + \bar{g}_{\rm Cl}V_{\rm Cl} + \bar{g}_{\rm Na}V_{\rm Na}}{\bar{g}_{\rm K} + \bar{g}_{\rm Cl} + \bar{g}_{\rm Na}} \approx \frac{\bar{g}_{\rm K}V_{\rm K} + \bar{g}_{\rm Cl}V_{\rm Cl} + \bar{g}_{\rm Na}V_{\rm Na}}{\bar{g}_{\rm K}} \ , \qquad \text{(S14)}$$

where $\bar{g}_{\rm Na}$, $\bar{g}_{\rm K}$, and $\bar{g}_{\rm Cl}$ are the channel conductance at the steady state. In our model, $D_{\rm Cl}$ and $D_{\rm Na}$ are modulated by DFs of 8-Br-cGMP. Due to small changes of $V$ at the resting potential, the MP upon 8-Br-cGMP stimulation obeys the following linearly approximated formula,

$$\hat{V} = V_{\rm K} - \eta_{\rm Cl}A_{\rm Cl}D_{\rm Cl} + \eta_{\rm Na}A_{\rm Na}D_{\rm Na} \ , \qquad \text{(S15)}$$

where $D_{\rm Cl}$ and $D_{\rm Na}$ are the normalized change of the effective densities of ClC and NaC induced by 8-Br-cGMP stimulation, respectively; they are one if their changes reach their maximum. The parameters, $V_{\rm K}$, $A_{\rm Cl} = \bar{g}_{\rm Cl}|V_{\rm Cl}|/\bar{g}_{\rm K}$, and $A_{\rm Na} = \bar{g}_{\rm Na}V_{\rm Na}/\bar{g}_{\rm K}$ depend on cell-dependent characteristics, and their values are specific for each cell. We disregarded the dependency of $D_{\rm Cl}$ and $D_{\rm Na}$ on the MP, as their affects are minuscule at the resting potential[10]. The values of both $\eta_{\rm Cl}$ and $\eta_{\rm Na}$ are one in the control condition and zero when the channel functions are blocked: ClC is blocked by 4,4'-dinitro-2,2'-stilbene-disulfonic acid (DNDS, 100 μM, n=5) and NaC is blocked by saxitoxin (STX, 10 nM, n=4). (**Fig. 1d**; **Supplementary Fig. 2 & 5**). In **Fig 4b**, $\eta_{\rm Cl}$ and $\eta_{\rm Na}$ for each MPTS were randomly set to one or zero regardless of actual experimental conditions.

**Calculation of model evaluation criterion**

According to Ogata's method[13,14], we approximated the log-evidence with the Markov Chain Monte Carlo (MCMC)-based posterior parameters plugged-in, where the logarithm Bayesian evidence (Eq. (2) in the text), $\ln E$, is formulated as follows,

$$\ln E = \sum_{l=1}^{L-1} \log \sum_{b=1}^{B} \exp\left\{(\beta_{l+1} - \beta_l)\sum_{i=1}^{I} \log p\left(V_i \middle| \Phi_i^{b,l}\right)\right\} - (L-1)\log B \quad , \qquad \text{(S16)}$$

where $\Phi_i^{b,l} = \{\phi_i^{b,l}, \theta^{b,l}\}$ represents the $b$-th set of parameter samples from MCMC simulation (total $B > 10^5$) and $\beta_l$ is the parameters controlling the range of MCMC parameter samples (inverse temperature) used in the Ogata's method ($L = 16$). We set these to $0 = \beta_1 < \beta_2 < \cdots < \beta_L = 1$ with equal intervals.

A simple MCMC algorithm such as Metropolis-Hasting[15,16] tends to accept proposals from regions of high probability density rather than from the entire space where the posterior density resides. Such an inadequacy in the sampling process may make marginalization practically inaccurate. Ogata's method[13,14] attempts to solve this practical issue by defining a modified marginal likelihood function weighted by an inverse temperature $\beta$,

$$z(\beta) = \int p(D|\Phi)^{\beta} p(\Phi) d\Phi \ , \qquad \text{(S17)}$$

where $D$ is data set, $z(1)$ is equivalent to the marginal likelihood (evidence), and $z(0) = 1$. From these observations, it is apparent that the marginal likelihood, $p(x) = z(1)$, is simply given by the product of quotients, each given by two adjacent modified marginal likelihood values as,

$$z \times p(D) = \frac{z(\beta_L)}{z(\beta_{L-1})} \times \cdots \times \frac{z(\beta_2)}{z(\beta_1)} = \prod_{l=1}^{L-1} \frac{z(\beta_{l+1})}{z(\beta_l)} \ . \qquad \text{(S18)}$$

Using the definition of Eq. (S17), the fraction term can be transformed as,

$$z\frac{z(\beta_{l+1})}{z(\beta_l)} = \int p(D|\Phi)^{\beta_{l+1}-\beta_l} \frac{p(D|\Phi)^{\beta_l} p(\Phi)}{z(\beta_l)} d\Phi = \int p(D|\Phi)^{\beta_{l+1}-\beta_l} p(\Phi|D;\beta_l) d\Phi \ . \qquad \text{(S19)}$$

The most right-hand side can be approximated as the mean of $p(D|\Phi^{b,l})^{\beta_{l+1}-\beta_l}$, with the $b$-th MCMC sample for the modified posterior with the $l$-th inverse temperature $\beta_l$, $\Phi^{b,l}$. Therefore, Eq. (S18) can be computed as,

$$p(D) = \prod_{l=1}^{L-1} \frac{1}{B}\sum_{b=1}^{B} p(D|\Phi^{b,l})^{\beta_{l+1}-\beta_l}, \tag{S20}$$

with MCMC samples from the modified posterior. Logarithm of Eq. (S20) is used as a model evaluation criterion (**Fig. 3a**).

**Calculation of model validation criterion**

We employed the expected error of single time point for the $i$-th MPTS,

$$e(i) = \frac{1}{T_i}\frac{1}{B}\sum_{b=1}^{B} \log\ p\left(V_i|\phi_i^{(b)}, \theta^{MAP}\right)\ , \tag{S21}$$

where $\theta^{MAP}$ is the typical core system parameter which is estimated as the mean values of MCMC samples obtained in the model evaluation step. Thus, the model validation criteria for all the validation data is defined as

$$e = \frac{1}{N}\sum_{i=1}^{N} e(i)\ , \tag{S22}$$

where $N$ is the number of MPTS samples.

**Leave one out cross validation (LOO-CV) algorithm**

Leave one out cross validation is performed as the following algorithm with $B \geq 10^5$:

For $i$ = 1 : (number of data sets)

1. Let LOO$(i)$ a data set omitting the $i$-th data
2. Obtain MCMC samples $\{\phi_i, \theta\}$, b = 1:B using LOO$(i)$
3. Obtain MCMC samples $\{\phi_i\}$, b = 1:B using mean a posteriori (MAP) parameter $\theta^{MAP}$ and $i$-th data $V_i$
4. Calculate Fitness (Log-likelihood) for $V_i$ by Eq. (S21).

Finally, total leave one out fitness is calculated as Eq. (S22).

**Calculation of likelihood for Netrin-1-induced MP**

We introduced the parameter, $S_{in}$, as the pseudo maximum concentration of 8-Br-cGMP by the model using the core system parameters estimated from 10 μM 8-Br-cGMP stimulation for the netrin-1-induced MPTS data set ($V_{netrin}$). When the netrin-1-induced MPTS and the system parameters are given, the posterior probability distribution of $\phi$ and $S_{in}$ becomes

$$p(\phi, S_{in}|V_{netrin}, \theta^{MAP}) \propto p(V_{netrin}|\phi, S_{in}, \theta^{MAP})p(\phi|\theta^{MAP})p(S_{in})\ , \tag{S23}$$

where prior, $p(S_{in})$, is a non-negative uniform distribution. To compare likelihoods, we computed the likelihood of MPTS induced by 5 μM 8-Br-cGMP ($V_{5\mu M}$) as the reference, and formulated the posterior as,

$$p\left(\phi, S_{in}|V_{5\mu M}, \theta\right) \propto p\left(V_{5\mu M}|\phi, S_{in}, \theta\right)p(\phi)p(S_{in})\ . \tag{S24}$$

**Model for 8-Br-cGMP diffusion**

We modeled the 8-Br-cGMP permeation into the cytoplasm in a growth cone to convert the extracellular 8-Br-cGMP concentration (bath-applied) to its intracellular concentration. We utilized a common enzymatic reaction, in which the membrane permeated 8-Br-cGMPs are in two forms in the cytoplasm upon permeation through the plasma membrane: bounded to the target proteins and in free forms. The model considers the permeation of 8-Br-cGMP into the cytoplasm with a fixed rate (**Supplementary Fig. 6a**). The model also considers that the intracellular 8-Br-cGMP concentration decreases proportionally at a rate at which the target bounded 8-Br-cGMP increases. Thus, the intracellular 8-Br-cGMP concentration ($S_{in}$) is expressed by the following ODE,

$$\frac{dS_{in}}{dt} = \frac{aS_{bath}}{K + S_{bath}} - bS_{in}\ , \tag{S25}$$

where $S_{bath}$ is the extracellularly applied concentration that corresponds to substrate in the Michaelis-Menten equation. By taking a steady state condition ($dS_{in}/dt = 0$), the following relationship is obtained,

$$S_{in} = \frac{AS_{bath}}{K + S_{bath}} \ . \qquad \text{(S26)}$$

We set $A = a/b = 7.8$ and $K = 8.3$, which $S_{in}$ saturates at $S_{bath} = 100$ μM (**Supplementary Fig. 6b**). These parameters were estimated to minimize the error between the model and experimental data (**Fig. 6b**).

## Supplementary Figure 1

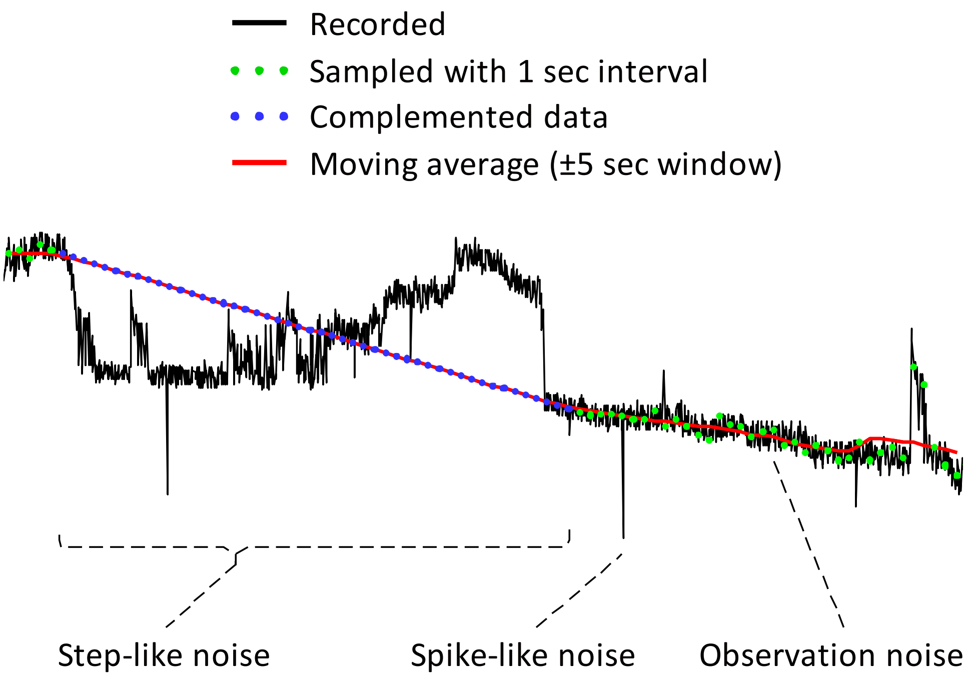


**Fig. 1.**
**Preprocessing of membrane potential time series**

The recorded raw data (black) was sampled at one sec intervals (green) to remove most of spike-like noise. The step-like noise, which was manually detected, was replaced with a straight line (blue). To estimate the size of the observation noise, the sampled data (green) was smoothed by applying a moving average filter with a ± 5 sec window (red), and the differences between the sampled and the smoothed at the same time point were obtained (see Methods in the text).

## Supplementary Figure 2

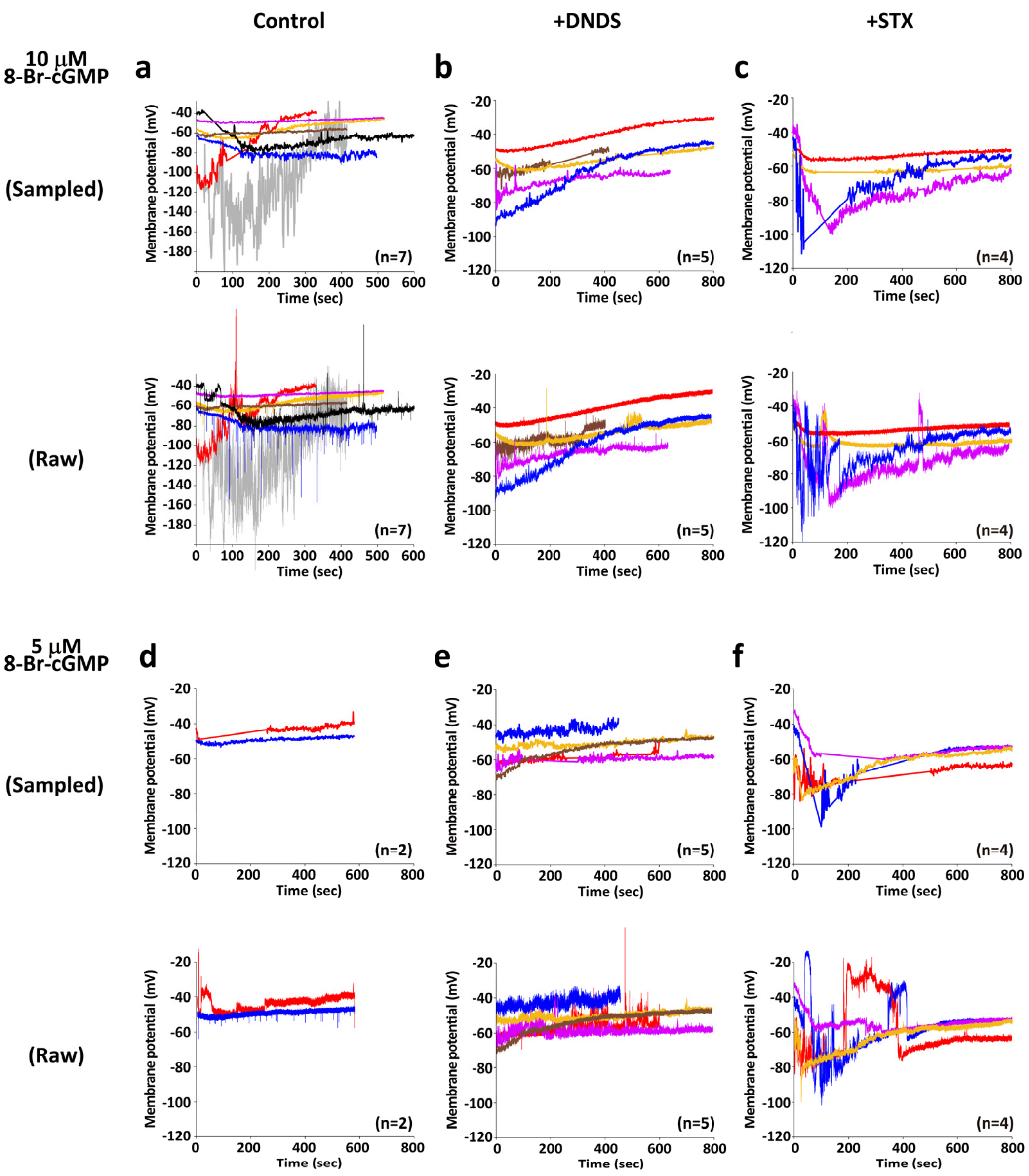


**Fig. 2.**
**MPTS recorded from growth cone of *Xenopus* spinal neurons**

(**a-b**) MPTS induced by 10 μM 8-Br-cGMP injected from a pipette under control conditions (a) (same as **Fig. 1b**), in the presence of the chloride channel inhibitor, DNDS, (**b**) and sodium channel inhibitor, STX, (**c**). (**d-f**) MPTS induced by 5 μM 8-Br-cGMP under control conditions (**d**), DNDS (**e**), and STX (**f**). Sampled and raw MPTSs are in the upper and lower rows, respectively.

## Supplementary Figure 3

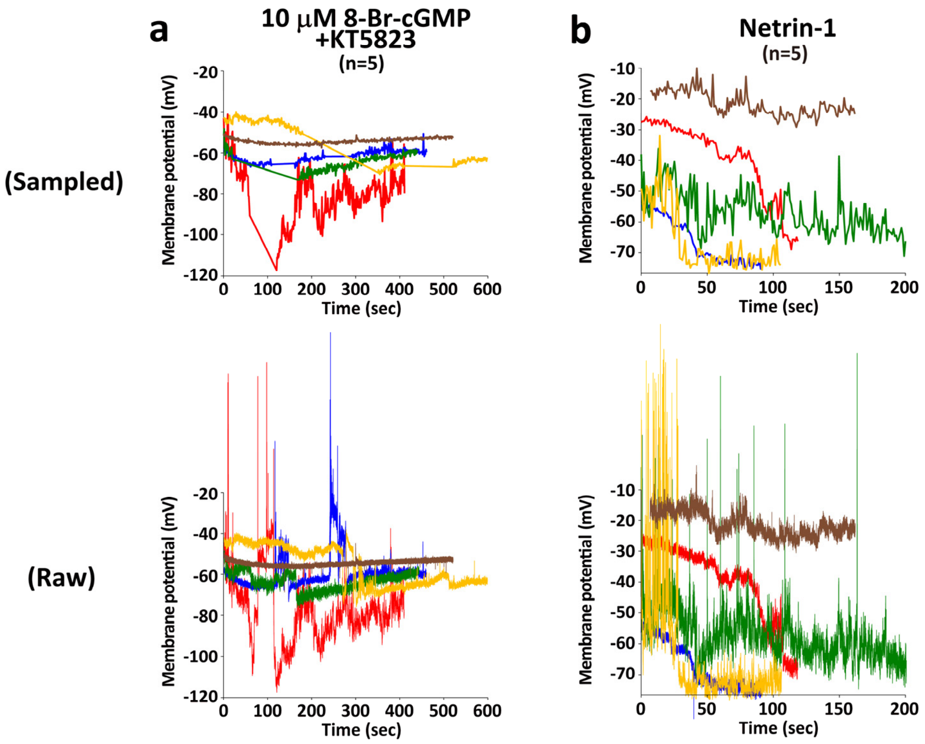


**Fig. 3.**
**MPTS induced under different conditions**

(**a**) MPTS induced by 10 μM 8-Br-cGMP in the presence of PKG inhibitor (KT5823). (**b**) MPTS induce by Netrin-1.

## Supplementary Figure 4

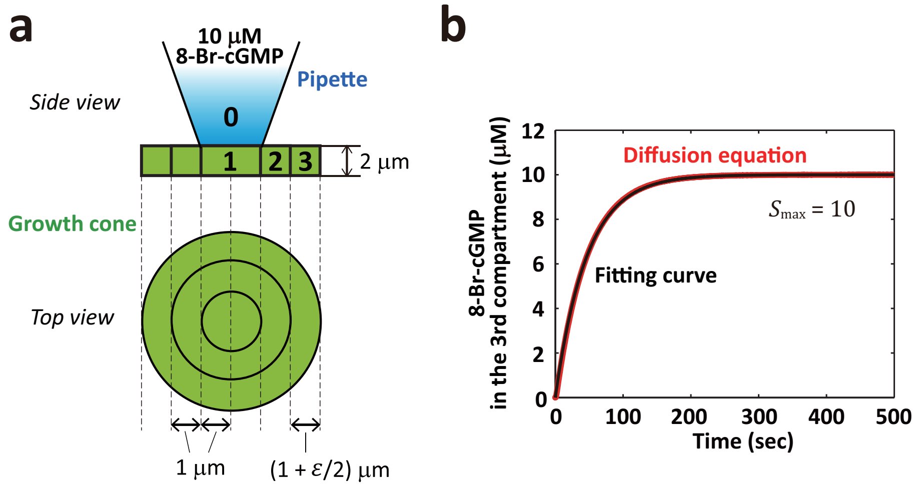


**Fig. 4.**
**Computation of the 8-Br-cGMP diffusion rate**

(**a**) Growth cone model for computing the time series of diffusion of 8-Br-cGMP, which is composed of three shell-shaped compartments. The 0-th compartment is the pipette, which releases 10 μM 8-Br-cGMP. To express the size variation of growth cone, Gaussian noise was added to the thickness of the outmost shell compartment (compartment #3). (**b**) The time series of 8-Br-cGMP concentration in the compartment #3 (red line) as calculated by the diffusion equation (Eq. (S2) in **Supplementary Methods**). The black line is the exponential function (Eq. (S1) in **Supplementary Methods**) fitted to the red line.

## Supplementary Figure 5

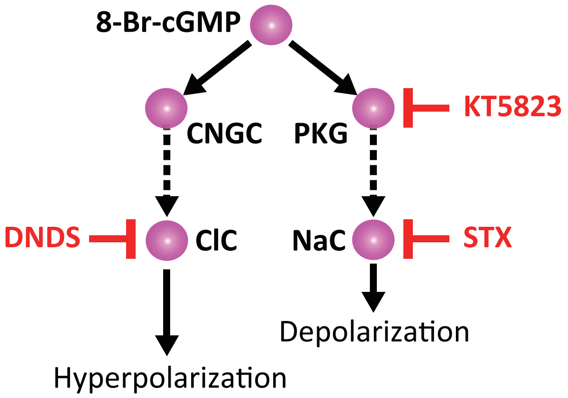


**Fig. 5.**
**Pharmacological application during MP induction**

DNDS, STX, and KT5823 inhibit specific molecules, chloride channel (ClC), sodium channel (NaC), and PKG, respectively.

## Supplementary Figure 6

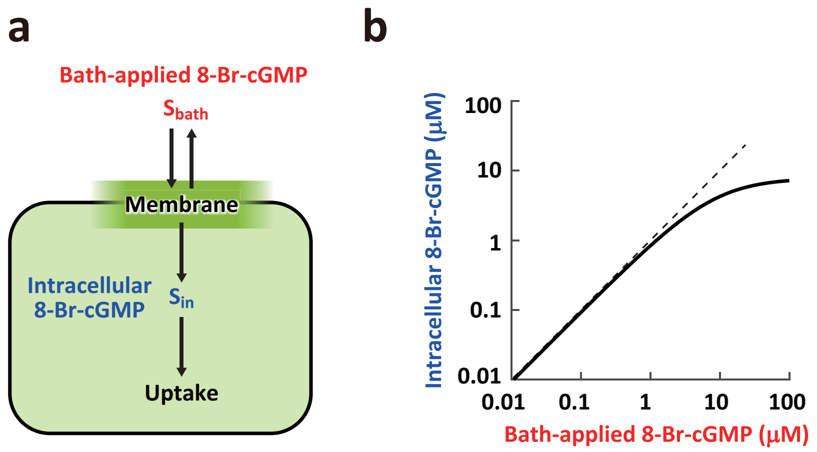


**Fig. 6.**
**Model for 8-Br-cGMP permeation into the cytoplasm via plasma membrane**

(**a**) Bath-applied extracellular 8-Br-cGMPs permeates through the plasma membrane into the cytoplasm with a fixed diffusion rate. Simultaneously, the level of the intracellular free 8-Br-cGMPs decreases as the uptake by bio-molecular reactions increases. (**b**) Computed intracellular 8-Br-cGMP concentration using the permeation model in (**a**).

## Supplementary Figure 7

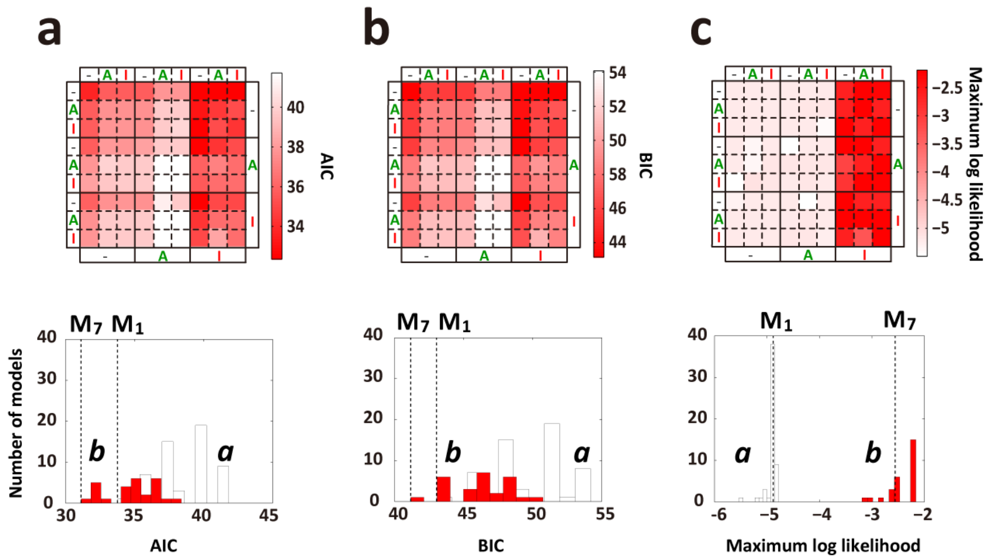


**Fig. 7.**
**Comparison of model selection criteria**

(**a**) Akaike Information Criterion (AIC). (**b**) Bayesian Information Criterion (BIC). (**c**) Maximum log likelihood. When Maximum log likelihood is denoted by $L$, AIC = $-2L + 2k$, BIC = $-2L + k\ln(n)$, respectively, where $k$ is the number of core system parameters of the corresponding model and $n$ is the number of data (=16). Upper panels show matrix representation for each criterion and lower panels show corresponding histogram as in **Fig. 3 a** and **b**.

## Supplementary Figure 8

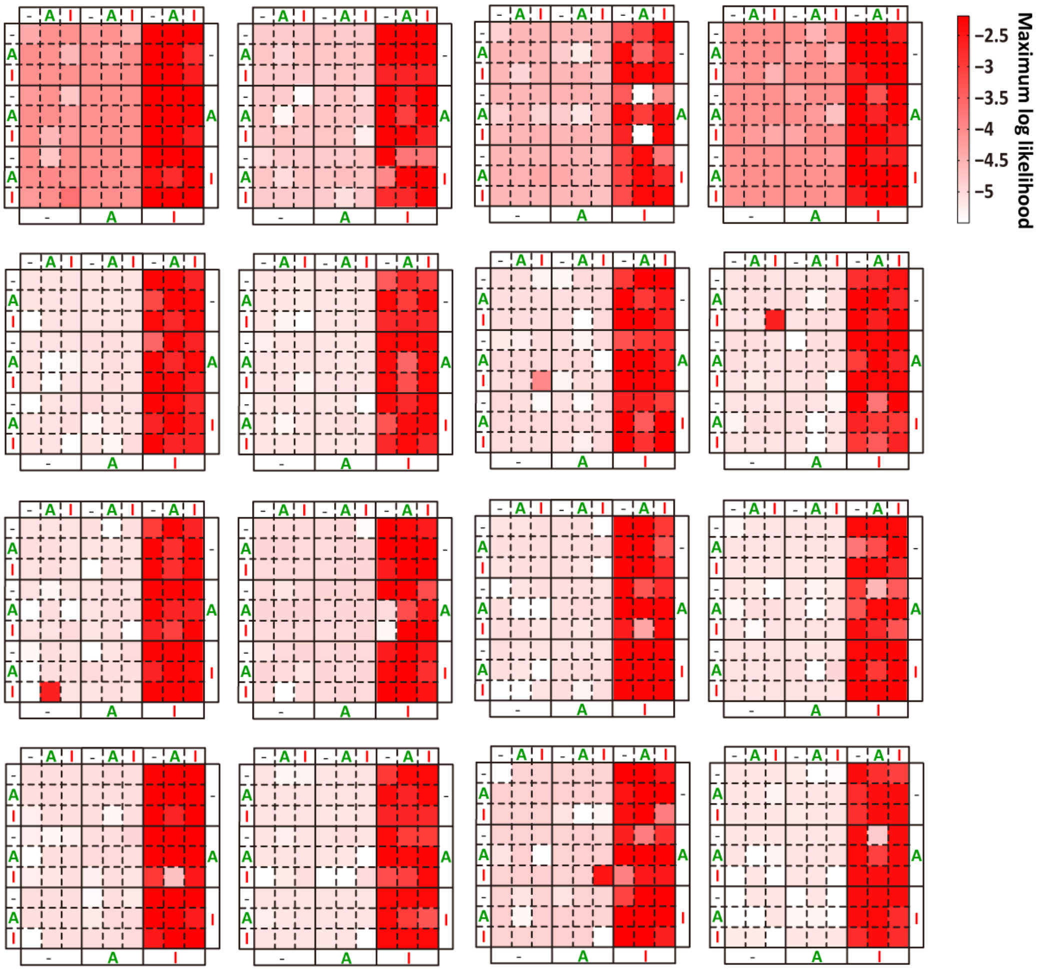


**Fig. 8.**
**Maximum log likelihood of each core system training step during leave one out (LOO) procedure**

Matrix representation of maximum log likelihood of each core system training step in LOO cross validation. Each panel shows the maximum log likelihood for one of the LOO dataset combination.

## Supplementary Figure 9

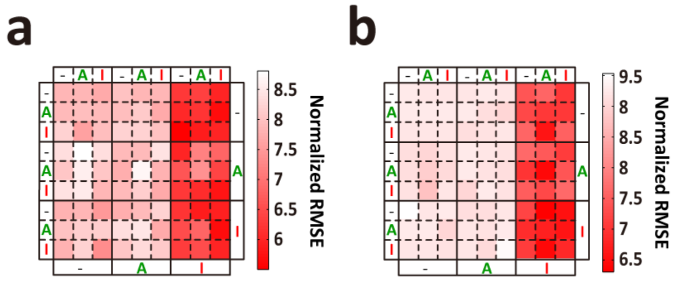


**Fig. 9.**
**Normalized RMSE of model predictability of untrained 8-Br-cGMP-induced MPTS**

(**a**) Matrix representation of normalized root mean squared error (RMSE) corresponding to **Fig. 5c**. (**b**) Same as **a**, but 5 μM 8-Br-cGMP MPTS was predicted corresponding to **Fig. 5d**.

## Supplementary Table 1
## Summary of prior distributions of model parameters.

Parameter types; $\theta$ (core system parameter), $\phi$ (peripheral parameter), and $c$ (experimental condition). Distributions; $N$ (Gaussian), $N_+$ (left-truncated Gaussian), and $N_+/N_+$ (left-truncated standard Cauchy distribution). Asterisk ($*$) represents $f$, $b$, $X$, or $Y$. The equation numbers are as indicated in **Supplementary Methods**.

| Parameter | Description | Unit | Equation | Type | Distribution | $\mu$ | $\sigma$ |
|---|---|---|---|---|---|---|---|
| $\tau_S$ | Time constant | Sec | (S1) | $\phi$ | $N_+$ | 0 | 100[#1] |
| $n, m$ | Hill coefficient | - | (S3), (S4) | $\theta$ | $N_+$ | 0 | 1[#2] |
| $k_{Xf*}/k_{Xf0}$<br>$k_{Xb*}/k_{Xf0}$ | Normalized reaction rates of $X$ | -<br>$(\mu M)^n$ | (S5) | $\theta$ | $N_+/N_+$ | - | - |
| $k_{Yf*}/k_{Yf0}$<br>$k_{Yb*}/k_{Yf0}$ | Normalized reaction rates of $Y$ | -<br>$(\mu M)^m$ | (S6) | $\theta$ | $N_+/N_+$ | - | - |
| $k_{Cl*}, k_{Na*}$ | Reaction rates of ion channel density, $D_{Cl}$ and $D_{Na}$ | sec$^{-1}$ | (S9)-(S12) | $\theta$ | $N_+$ | 0 | 1[#3] |
| $A_{Cl0}$ | Mean of $A_{Cl}$[#4] | mV | - | $\theta$ | $N_+$ | 0 | 10[#5] |
| $A_{Na0}$ | Mean of $A_{Na}$[#4] | mV | - | $\theta$ | $N_+$ | 0 | 10[#5] |
| $A_{Cl}$ | Maximum MP shift by ClC | mV | (S15) | $\phi$ | $N_+$ | $A_{Cl0}$ | 5[#5] |
| $A_{Na}$ | Maximum MP shift by NaC | mV | (S15) | $\phi$ | $N_+$ | $A_{Na0}$ | 5[#5] |
| $V_K$ | Reversal potential of $K^+$ channel | mV | (S15) | $\phi$ | $N$ | $-60$[#5] | 5[#5] |
| **Parameter** | **Description** | **Unit** | **Equation** | **Type** | **Value** | | |
| $S_{max}$ | Maximum 8-Br-cGMP conc. | μM | (S1) | $c$ | 10 or 5 | | |
| $\eta_{Cl}$ | DNDS application | - | (S15) | $c$ | 0 (applied) or 1 (control) | | |
| $\eta_{Na}$ | STX application | - | (S15) | $c$ | 0 (applied) or 1 (control) | | |

#1. Estimated from Monte Carlo simulation in **Supplementary Fig. 4**.
#2. We set the prior distributions for $n$ and $m$ to the left-truncated Gaussians, $N(0,1^2)$, whose s.d. is smaller than the number of binding sites of cGMP to CNGC and PKG (4 and 4, respectively)[3,17], because the effective Hill coefficient of the *in vivo* condition estimated to be small due to molecular crowding[18].
#3. The time constants for the MP shift can be more than a second according to the data (**Supplementary Fig. 2 and 3**).
#4. According to the MPTS (**Supplementary Fig. 2**), the maximum MP shifts by ClC ($A_{Cl}$; $\phi$) and NaC ($A_{Na}$; $\phi$) depends on each cell. Thus, we considered their values ($A_{Cl0}$ and $A_{Na0}$) are cell-independent, and we categorized the system parameters ($\theta$).
#5. The variation in resting potential comes from the reversal potential of the $K^+$ channel ($V_K$) and variations of the mean and s.d. of the contributions of ClC and NaC to MP ($A_{Cl0}$, $A_{Na0}$, $A_{Cl}$, and $A_{Na}$). The mean of reversal potential of the potassium channel ($V_K$; $\phi$) was set to $-60$ mV according to the initial values of experimental MPTS (**Supplementary Fig. 2**)[2]. Because the largest difference between the initial values of the time series is about 40 mV, we distributed this difference to variations of $V_K$, $A_{Cl0}$, $A_{Na0}$, $A_{Cl}$, and $A_{Na}$ by setting their s.d. to 5, 10, 10, 5, and 5, respectively.

## References


1 Koutalos, Y., Brown, R., Karpen, J. & Yau, K. Diffusion coefficient of the cyclic GMP analog 8-(fluoresceinyl)thioguanosine 3',5' cyclic monophosphate in the salamander rod outer segment. *Biophys J* **69**, 2163-2167 (1995).
2 Nishiyama, M., von Schimmelmann, M. J., Togashi, K., Findley, W. M. & Hong, K. Membrane potential shifts caused by diffusible guidance signals direct growth-cone turning. *Nat Neurosci* **11**, 762-771 (2008).
3 Kaupp, U. B. & Seifert, R. Cyclic nucleotide-gated ion channels. *Physiol Rev* **82**, 769-824, doi:10.1152/physrev.00008.2002 (2002).
4 Togashi, K. *et al.* Cyclic GMP-gated CNG channels function in Sema3A-induced growth cone repulsion. *Neuron* **58**, 694-707 (2008).
5 Duran, C., Thompson, C. H., Xiao, Q. & Hartzell, H. C. Chloride channels: often enigmatic, rarely predictable. *Annu Rev Physiol* **72**, 95-121, doi:10.1146/annurev-physiol-021909-135811 (2010).
6 Boudes, M. & Scamps, F. Calcium-activated chloride current expression in axotomized sensory neurons: what for? *Frontiers in molecular neuroscience* **5**, 35, doi:10.3389/fnmol.2012.00035 (2012).
7 Li, Z., Zhang, G., Feil, R., Han, J. & Du, X. Sequential activation of p38 and ERK pathways by cGMP-dependent protein kinase leading to activation of the platelet integrin alphaIIb beta3. *Blood* **107**, 965-972, doi:10.1182/blood-2005-03-1308 (2006).
8 Mikami, M. & Yang, J. Short hairpin RNA-mediated selective knockdown of NaV1.8 tetrodotoxin-resistant voltage-gated sodium channel in dorsal root ganglion neurons. *Anesthesiology* **103**, 828-836 (2005).
9 Hudmon, A. *et al.* Phosphorylation of sodium channel Na(v)1.8 by p38 mitogen-activated protein kinase increases current density in dorsal root ganglion neurons. *J Neurosci* **28**, 3190-3201, doi:10.1523/JNEUROSCI.4403-07.2008 (2008).
10 Hodgkin, A. L. & Huxley, A. F. A quantitative description of membrane current and its application to conduction and excitation in nerve. *J Physiol* **117**, 500-544 (1952).
11 Fall, C. P., Marland, E. S., Wagner, J. M. & Tyson, J. J. *Computational Cell Biology*. (Springer, 2002).
12 Ferrell, J. E., Jr. & Machleder, E. M. The biochemical basis of an all-or-none cell fate switch in Xenopus oocytes. *Science* **280**, 895-898 (1998).
13 Ogata, Y. A Monte Carlo method for an objective Bayesian procedure. *Ann. Inst. Statist. Math.* **42**, 403-433 (1990).
14 Nagata, K., Sugita, S. & Okada, M. Bayesian spectral deconvolution with the exchange Monte Carlo method. *Neural Netw* **28**, 82-89, doi:10.1016/j.neunet.2011.12.001 (2012).
15 Metropolis, N., Rosenbluth, A. W., Rosenbluth, M. N., Teller, A. H. & Teller, E. Equations of State Calculations by Fast Computing Machines. *Journal of Chemical Physics* **21**, 1087-1092 (1953).
16 Hastings, W. K. Monte Carlo Sampling Methods Using Markov Chains and Their Applications. *Biometrika* **57**, 97-109 (1970).
17 Smith, J. A., Reed, R. B., Francis, S. H., Grimes, K. & Corbin, J. D. Distinguishing the roles of the two different cGMP-binding sites for modulating phosphorylation of exogenous substrate (heterophosphorylation) and autophosphorylation of cGMP-dependent protein kinase. *J Biol Chem* **275**, 154-158 (2000).
18 Aoki, K., Yamada, M., Kunida, K., Yasuda, S. & Matsuda, M. Processive phosphorylation of ERK MAP kinase in mammalian cells. *Proc Natl Acad Sci U S A* **108**, 12675-12680, doi:10.1073/pnas.1104030108 (2011).
